# Supplementary material for: PAF1c links S-phase progression to immune evasion and MYC function in pancreatic carcinoma
Source: Nat Commun. 2024 Feb 16;15:1446. doi: 10.1038/s41467-024-45760-8 (PMC10873513; doi:10.1038/s41467-024-45760-8)

Supplementary Figure 1

a

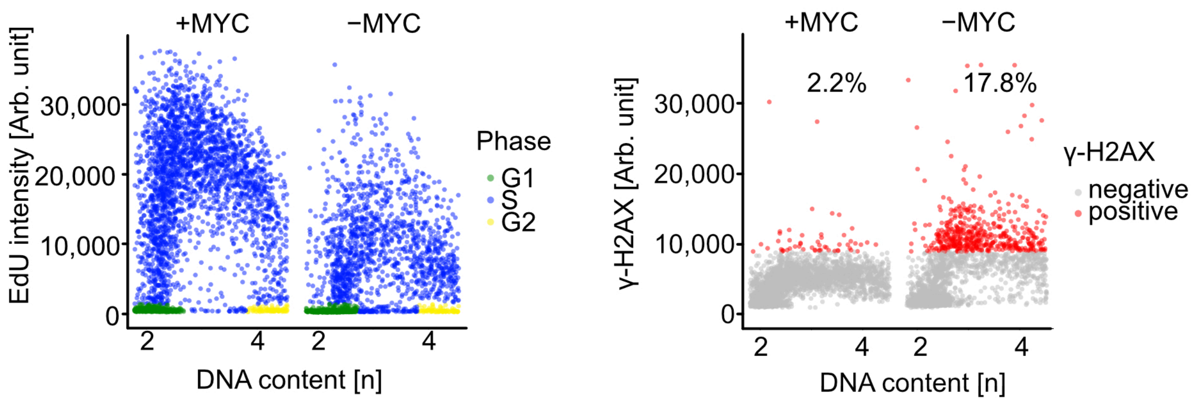

b

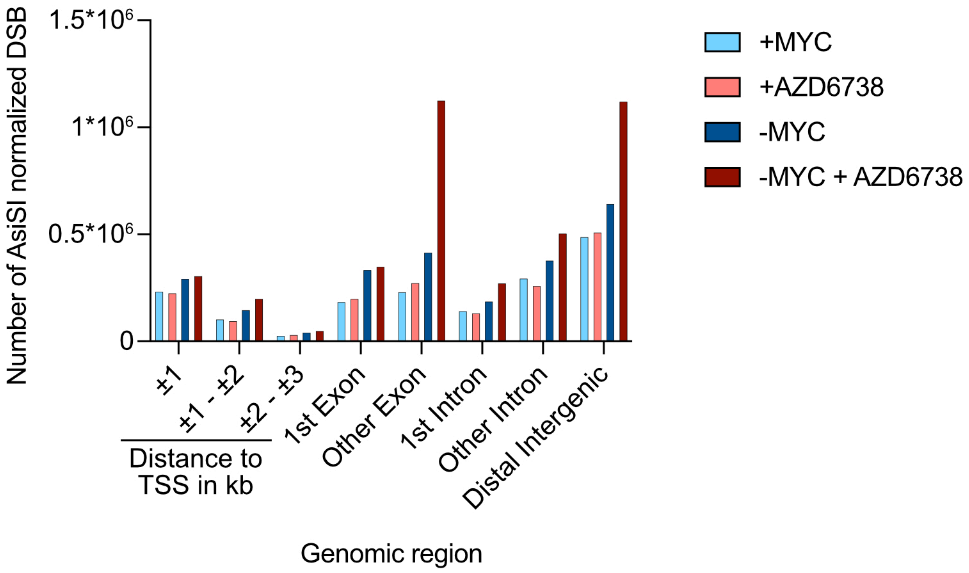

Supplementary Figure 1. Characterization of MYC depletion.

a. Quantitative image-based cytometry showing DNA content on the x-axis and y-axis measuring EdU incorporation. Left panel shows gates for different cell cycle phases, right panel shows the threshold for considering γ-H2AX positive cells. These plots are related to the experiment shown in Figure 1b-d (n=3 independent experiments).

b. Bar plot showing the number of AsiSI-normalized reads, quantifying double-strand breaks at the indicated genomic regions. For this plot, data from the three biological replicates were pooled.

Source data are provided as a Source Data file.

Supplementary Figure 2

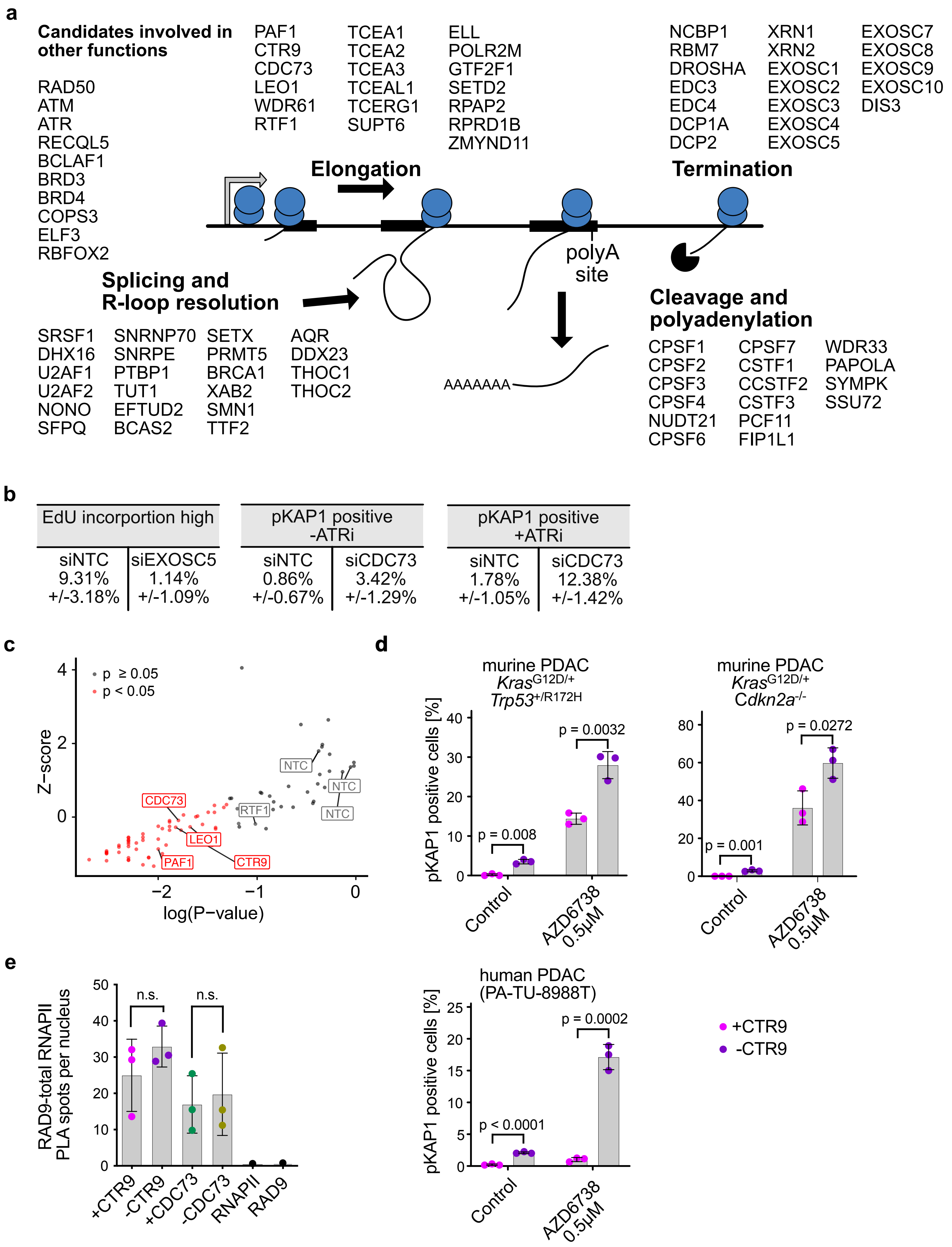

Supplementary Figure 2. Focused siRNA screen of genes involved in S-phase progression.

a. Scheme illustrating siRNA targets and their function.

b. Quantification of data shown in Fig. 2a. The mean value and the standard deviation are shown (n=3 independent experiments, except for “+ ATRi”, n=2).

c. Scatter plot illustrating hits from the “EdU incorporation” read-out. Each dot represents an siRNA. Z-score was calculated based on the number of EdU-high cells. P values were calculated using unpaired t-test, two-sided. Hits with P value <0.05 are colored in red. Labeled are siRNAs targeting the components of the PAF1c and non-targeting control (NTC) (n=3 independent experiments; unpaired two-sided t-test).

d. Bar plots documenting percentage of pKAP1 positive cells in p53-mutant (top left) or p16ink4a- deficient (top right) murine PDAC cells as well as PA-TU-8988T (bottom) human PDAC cells. Murine PDAC cell lines express doxycycline-inducible shCTR9. PA-TU-8988T cells were transfected with siRNAs targeting CTR9. Treatment with AZD6738 (0.5 µM) was for 72h. Data are presented as mean ± s.d. (n=3 independent experiments; unpaired two-sided t-test).

e. Bar plots quantifying the number of nuclear PLA foci between RAD9 and total RNAPII. Data are presented as mean ± s.d. (n=3 independent experiments; unpaired two-sided t-test).

Source data are provided as a Source Data file.

Supplementary Figure 3

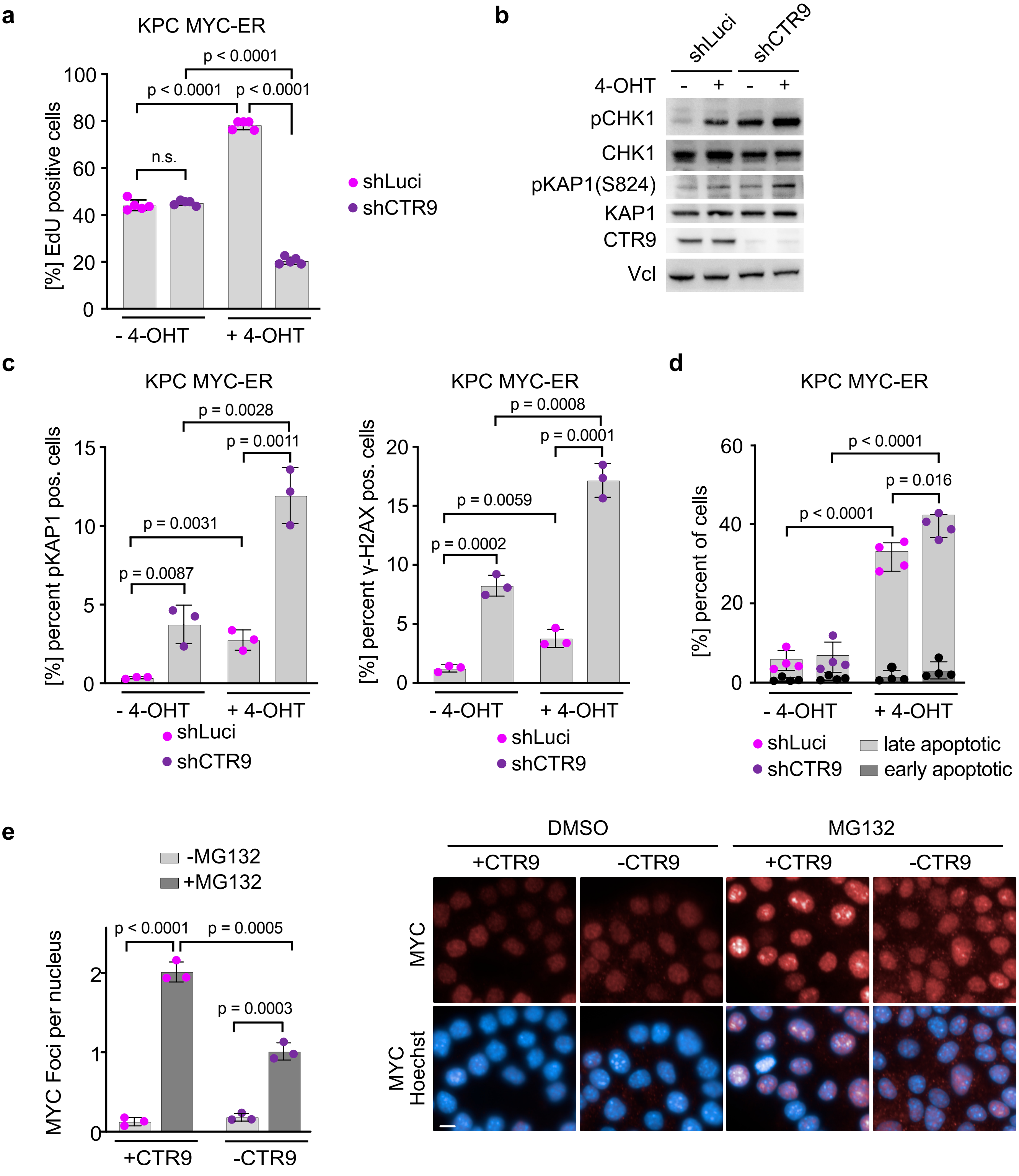

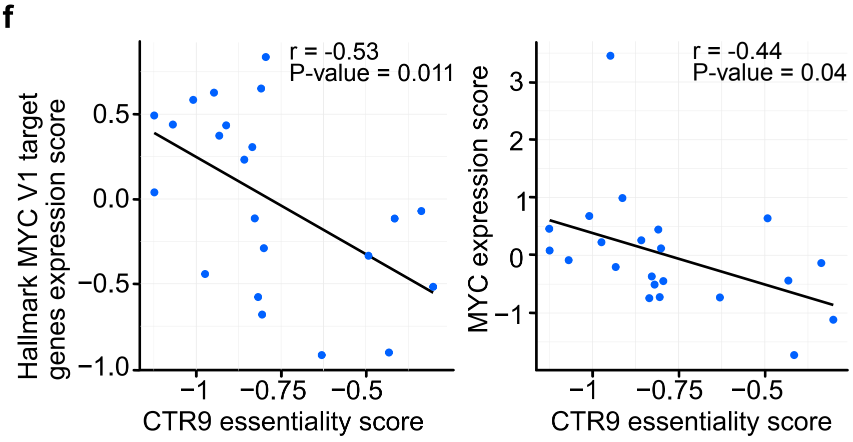

g

	Gene	r	P-value
PAF1c	*LEO1*	-0.59	0.004
PAF1c	*CDC73*	-0.45	0.038
PAF1c	*CTR9*	-0.44	0.04
	EXOSC5	-0.35	0.112
	CPSF2	-0.24	0.287
	AQR	-0.08	0.723
	ATR	-0.08	0.711
	XAB2	-0.05	0.823
	ACTB	0.004	0.985
	SUPT6H	0.22	0.323
	PCF11	0.26	0.24
	EFTUD2	0.38	0.083
	SFPQ	0.50	0.018

Supplementary Figure 3. MYC activation enhances the dependence on PAF1c.

a. Quantification of EdU positive cells in KPC MYC-ER cells expressing shRNA targeting CTR9. Where indicated, MYCER was activated by adding 4-hydroxytamoxifen (4-OHT; 200nM) for 24 h. Doxycycline was added for 48h, EdU was added 20 minutes before fixation. Data are presented as mean ± s.d. (n=5 independent experiments; unpaired two-sided t-test).

b. Immunoblot of the indicated proteins and phosphoproteins in KPC-MYCER cells documenting their levels upon CTR9 depletion and MYC activation. CTR9 depletion was induced by doxycycline addition for 72h and MYC activation was induced by the addition of 4-OHT for 24h (pCHK1: n=2 independent experiments; pKAP1: n=4 independent experiments).

c. Bar plots documenting percentage of pKAP1- (left) and γH2Ax-positive (right) cells under the experimental conditions as in (b). Data are presented as mean ± s.d. (n=3 independent experiments; unpaired two-sided t-test).

d. Annexin V/propidium iodide (PI) flow cytometry analysis measuring early (Annexin V+/propidium iodide-) and late (Annexin V+/propidium iodide+) apoptosis. shRNA expression was induced by doxycycline and, where indicated, MYCER was activated by the addition of 4-OHT, respectively, for 48 hours. Data are presented as mean ± s.d. (n=4 independent experiments; unpaired two-sided t-test).

e. Quantification of MYC multimers (foci). Nuclei were stained with Hoechst. CTR9 depletion was induced by doxycycline addition for 48h and MG132 (20 μM) for 4h before fixation as indicated. Data are presented as mean ± s.d. (n=3 independent experiments; unpaired two-sided t-test). Representative immunofluorescence pictures are shown on the right.

f. Correlation between Hallmark MYC target gene V1 (left) or MYC (right) expression scores and the essentiality score of CTR9. A lower essentiality score means that a gene is more likely to be dependent in a given cell line. Each graph includes the corresponding Pearson correlation coefficient and p-value for the regression analysis, in multiple pancreatic carcinoma cell lines.

g. Table showing Pearson correlation coefficient and p-value between MYC expression score and the dependency on the indicated genes in multiple human pancreatic carcinoma cell lines. Non-significant p-values are indicated in red.

Source data are provided as a Source Data file.

Supplementary Figure 4

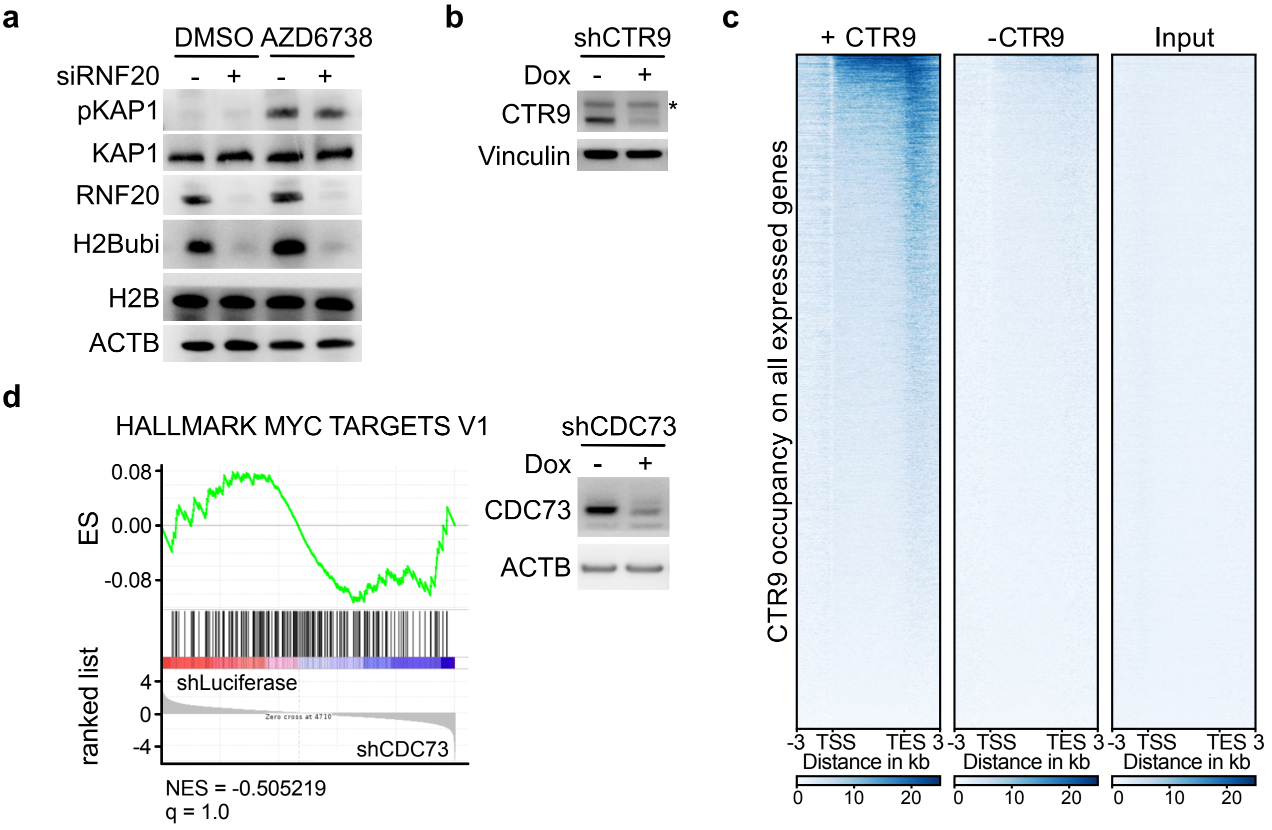

Supplementary Figure 4. Characterization of PAF1c function in PDAC cells.

a. Immunoblot documenting levels of the indicated proteins or posttranslational modifications 48hrs after siRNA-mediated depletion of RNF20 in KPC cells in the presence or absence of AZD6738 (0.2 μM; 72 h) (n=3 independent experiments).

b. Immunoblot of cells harboring doxycycline-inducible shRNA targeting CTR9. Doxycycline was added for 48h where indicated. * refers to a non-specific band. Vinculin was loading control (n=4 independent experiments).

c. Heat map of CTR9 occupancy for all expressed (10920) genes analyzed by ChIP-Rx in cells expressing shCTR9. Where indicated (“-CTR9”), doxycycline was added for 48h (n=2 independent experiments).

d. (Left): GSEA enrichment plot of MYC target gene set V1 in cells expressing shRNA targeting luciferase versus CDC73 (n=3). (Right): Immunoblot documenting depletion of CDC73(n=3 independent experiments).

Supplementary Figure 5

a

Gene	Length in kb	shCTR9		shCDC73		shMYC	
		log_2FC	FDR	log_2FC	FDR	log_2FC	FDR
Atr	94	-1.97	5.5E-18	-1.72	1.6E-11	-1.48	3.8E-13
Rad50	58	-1.61	2.9E-20	-1.69	3.3E-17	-0.49	3.6E-04
Fancd2	65	-1.49	2.6E-16	-1.84	1.5E-16	-0.74	3.2E-07
Mre11a	52	-1.26	3.0E-21	-1.25	3.6E-14	-0.86	1.1E-14
Fanci	58	-1.25	1.9E-10	-1.36	2.3E-09	-0.77	2.4E-06
Topbp1	45	-1.29	9.6E-29	-0.84	1.6E-07	-0.95	3.5E-24
Pms1	108	-1.21	3.6E-05	-1.85	3.0E-07	-0.72	1.1E-02
Blm	80	-0.59	3.7E-03	-1.17	8.8E-07	-1.53	2.6E-16

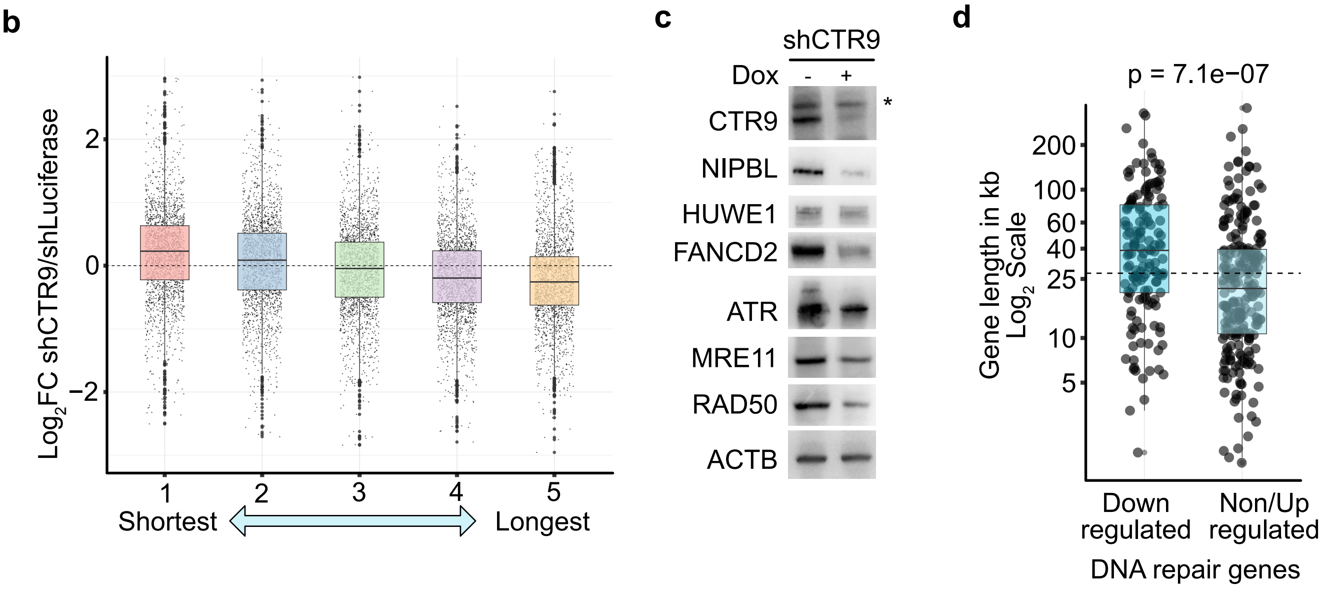

Supplementary Figure 5. Full-length transcription of long genes requires CTR9.

a. Table showing the length of genes involved in DNA repair and their change in expression upon depletion of CTR9, CDC73 or MYC. FDR is calculated to adjust P values for multiple comparisons (n=3 independent experiments).

b. Box plot showing log2FC of mRNA levels upon CTR9 depletion compared to control. Genes are shown as dots and are stratified based on their length from bin 1 (shortest) to 5 (longest), with bin 1,2,3 = 2066 genes, and bin 4,5 = 2065 genes. Log2FC was calculated using reads mapped to the second half of each gene (n=3 independent experiments). In the box plot, the central line shows the median and the borders of the boxes extend from the 25th to the 75th percentile, and the whiskers were plotted using the Tukey method.

c. Immunoblot documenting levels of DNA repair proteins upon depletion of CTR9. * refers to a non-specific band. CTR9 depletion was achieved by adding doxycycline for 48h (n=4 independent experiments).

d. Box plot showing gene length in kilobase (kb) comparing genes expressed in DNA repair GO term (GO-0006281, n=375 genes) stratified according to their differential expression upon CTR9 depletion to either down-regulated (n= 147 genes) or non/up regulated (n = 228 genes). The dashed black line represents the median of all expressed genes in the DNA repair GO term. P value was calculated using non-parametric Wilcoxon test. In the box plot, the central line shows the median and the borders of the boxes extend from the 25th to the 75th percentile, and the whiskers were plotted using the Tukey method.

Source data are provided as a Source Data file.

Supplementary Figure 6

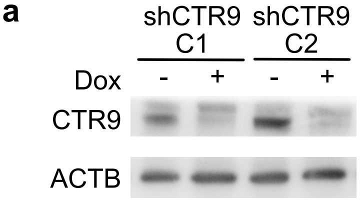

b

GO Term	FDR shCTR9	FDR shCDC73	FDR shMYC
antigen processing and presentation via MHC class Ib (GO:0002475)	1.72E-08	2.13E-06	2.10E-03
positive regulation of T cell mediated cytotoxicity (GO:0001916)	5.44E-08	8.66E-05	7.53E-04
regulation of T cell mediated cytotoxicity (GO:0001914)	9.92E-08	1.37E-04	1.35E-03

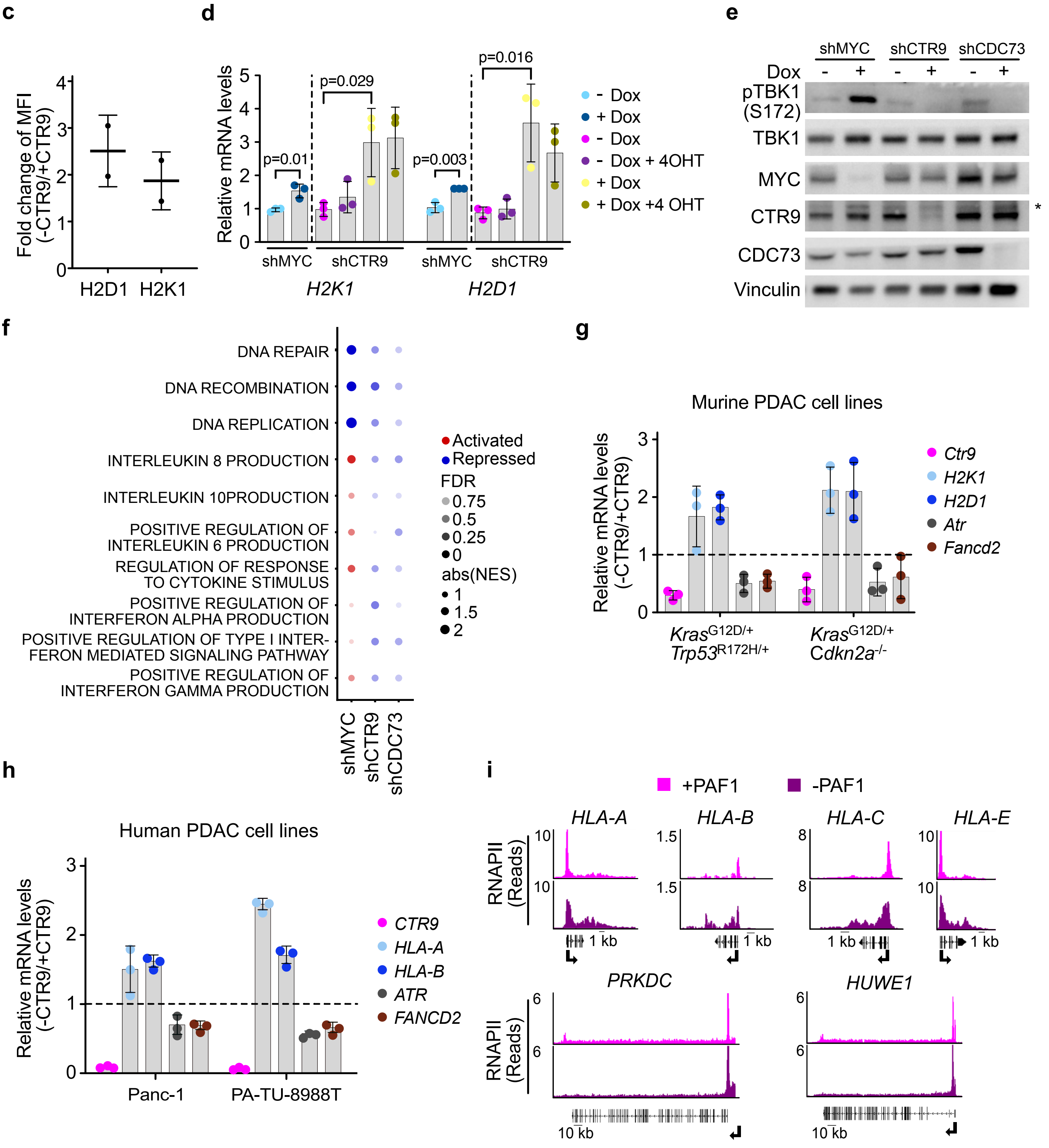

Supplementary Figure 6. PAF1c suppresses the expression of MHC class I genes.

a. Immunoblot documenting depletion of CTR9 in two different KPC clones harboring doxycycline-inducible shRNA targeting CTR9. * refers to a non-specific band.

b. Table showing FDR of upregulated GO terms upon depletion of CTR9, CDC73 or MYC (n=3 independent experiments).

c. Flow cytometry analysis of cell surface expression of H2-D1 and H2-K1 upon CTR9 depletion by doxycycline addition for 48 h. Shown is relative mean florescence intensity. Data are presented as mean ± s.d. (n=2 independent experiments).

d. RQ-PCR analysis of expression of the indicated MHC class I genes after shRNA-mediated depletion of MYC and after depletion of CTR9 in KPC-MYCER cells. Where indicated, MYCER was activated by adding 4-hydroxytamoxifen (4-OHT; 200nM) for 24 h. Doxycycline was added for 48h. Data are presented as mean ± s.d. (n=3 independent experiments; unpaired two-sided t-test). Only significant P values are shown.

e. Immunoblot for KPC cells harboring shRNA targeting either MYC, CTR9 or CDC73. Depletion was induced by doxycycline addition for 48 h. Vinculin was loading control.

f. Dot plot representing the GSEA analysis showing the response of genes of the indicated GO terms to depletion of either MYC, CTR9 or CDC73 using shRNA. The size of each dot represents the NES in either direction. The FDR is represented by the intensity of the color with darker color indicating more significance.

g. RT-qPCR measurement documenting the effects of CTR9 depletion in murine PDAC cells with the indicated genotypes. CTR9 depletion was induced by doxycycline addition for 48 h. Data are presented as mean ± s.d. (n=3 independent experiments).

h. Same as e, but for PA-TU-8988T and PANC1 human PDAC cell lines transfected with siRNA targeting CTR9 or non-targeting control (48 h). Data are presented as mean ± s.d. (n=3 independent experiments).

i. Browser track pictures showing RNAPII distribution over several MHC class I and DNA repair genes in human DLD1 cells expressing an auxin-degradable PAF1 allele.

Source data are provided as a Source Data file.

Supplementary Figure 7

a

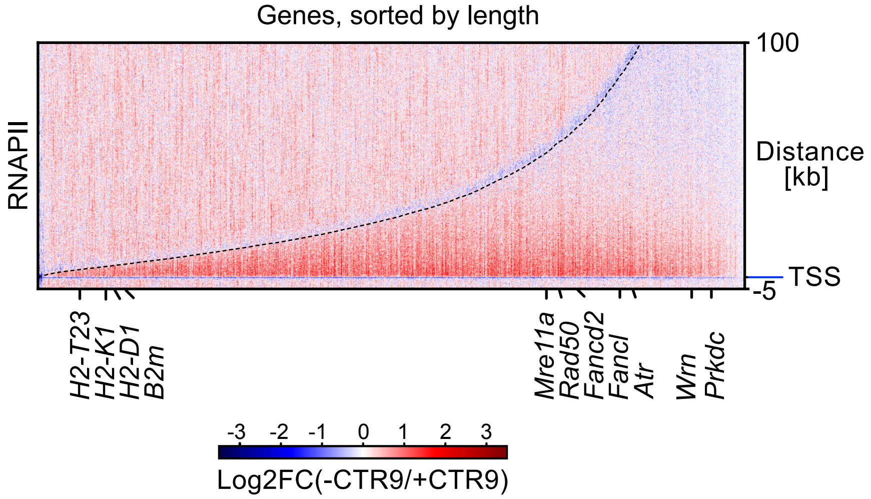

b

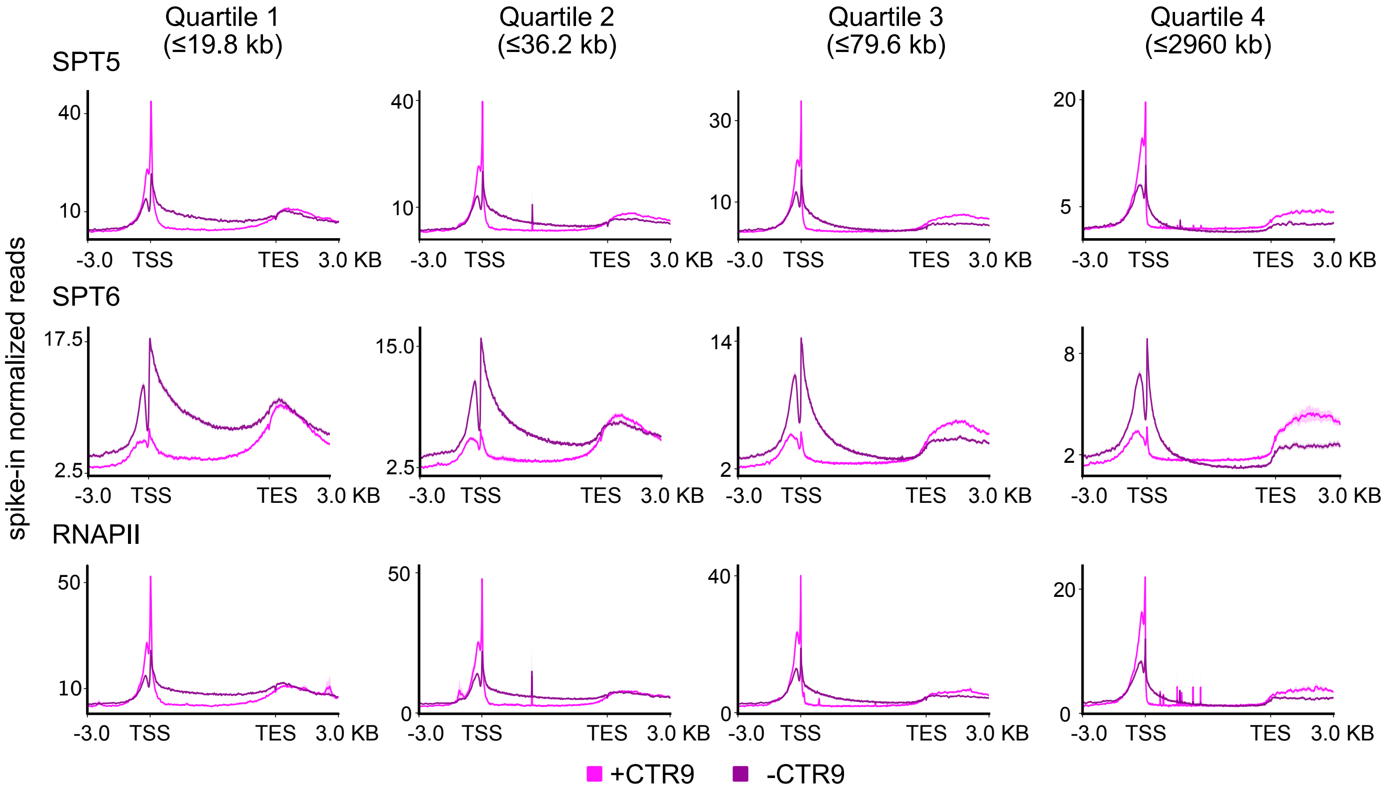

Supplementary Figure 7. Effects of CTR9 depletion on transcription elongation.

a. Heat map of log2fold change in RNAPII occupancy for all expressed (n=10920) genes analyzed by spike-normalized ChIP-sequencing (ChIP-Rx) in KPC cells expressing shCTR9 comparing CTR9 depleted samples to EtOH control. Genes are sorted according to their length from shortest (left) to longest (right). The black dashed line represents the end of the gene. Some genes belonging either to the DNA repair GO term or MHC class I GO term are indicated.

b. Metagene plots mapping SPT5, SPT6 and RNAPII chromatin occupancy by ChIP-Rx, shown for the same group of genes in Fig. 3g, stratified by increasing length from quartile 1 (shortest) to quartile 4 (longest), where each quartile consists of 2091 genes, except quartile 4 with 2092 genes (n=2 independent experiments).

Supplementary Figure 8

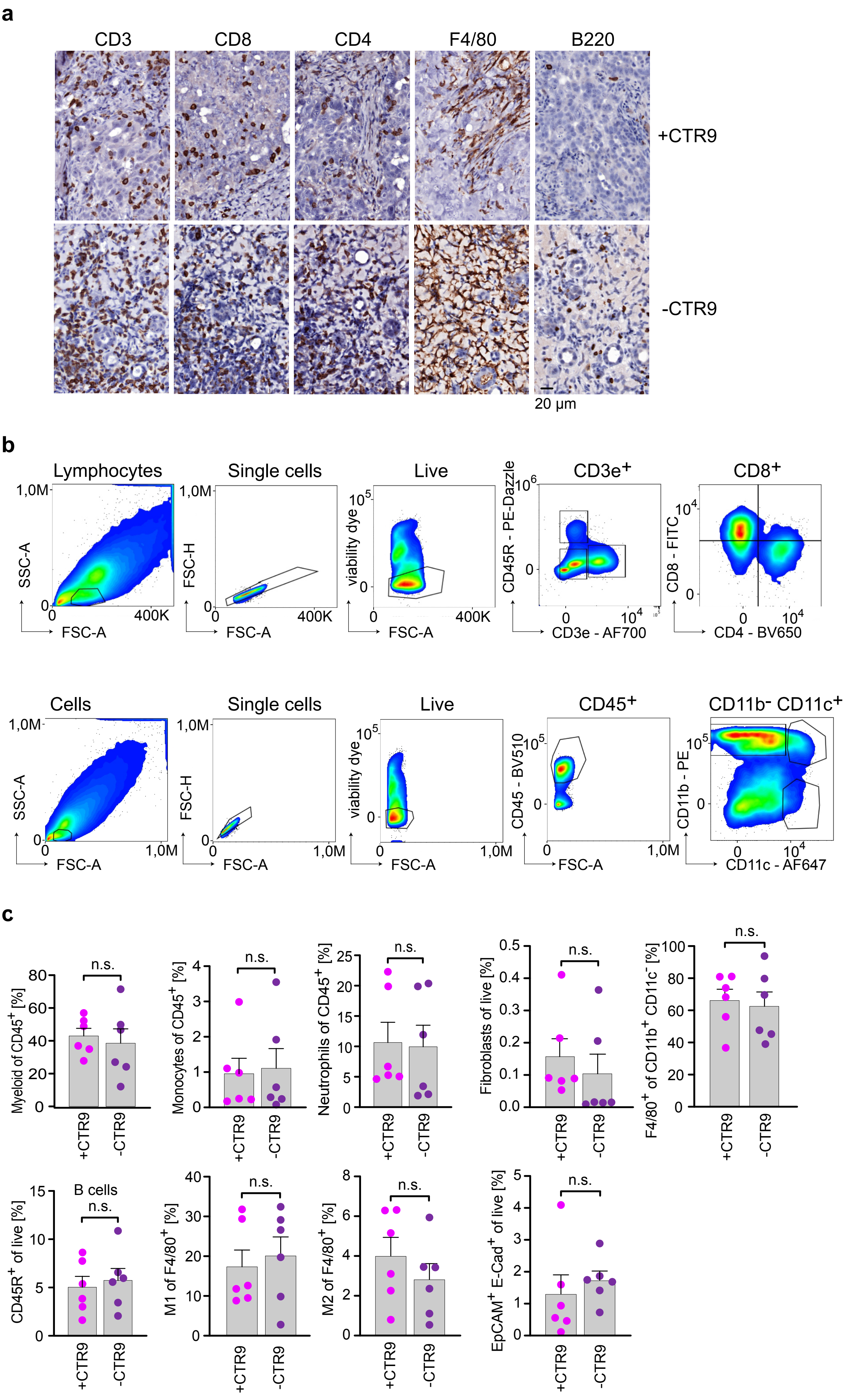

Supplementary Figure 8. Characterization of the tumor microenvironment after CTR9 depletion.

a. Representative images showing CD3, CD8, CD4 and B cells (B220) as well as macrophages (F4/80) stained in brown. Scale bar: 20μm.

b. Flow cytometry plots, documenting the gating strategy for the respective immune cell population.

c. Flow cytometry analysis of immune cell subsets in the tissues of tumors initiated by orthotopic transplantation of KPC cells harboring doxycycline-inducible shNTC or shCTR9. After 7 days of transplantation, doxycycline treatment was done for 3 days. Pre-gating was performed using single living cells. Cells were defined as cDC2 (CD45+, CD11b+, CD11c+), monocytes (CD45+, CD11b+, CD11c-, Ly6C+, Ly6G-), neutrophils (CD45+, CD11b+, CD11c, Ly6C-,Ly6G+), fibroblasts (CD45-,EpCAM-,E-cadherin-, CD31-, PDGFRα+, Pdpn+), macrophages (CD45+, F4/80+, CD11b+, CD11c-), B-cells (CD45+, CD3e-, CD45R+), M1 macrophage subtype (MHCII+, CD206-), M2 macrophage subtype (MHCII-, CD206+) and epithelial, likely tumor, cells (EpCAM+, E-cadherin+). Data are presented as mean ± s.d. (n=6 independent tumors; unpaired two-sided t-test).

Source data are provided as a Source Data file.

Uncropped immunoblot images of supplementary figures
Supplementary Figure 3 panel b.

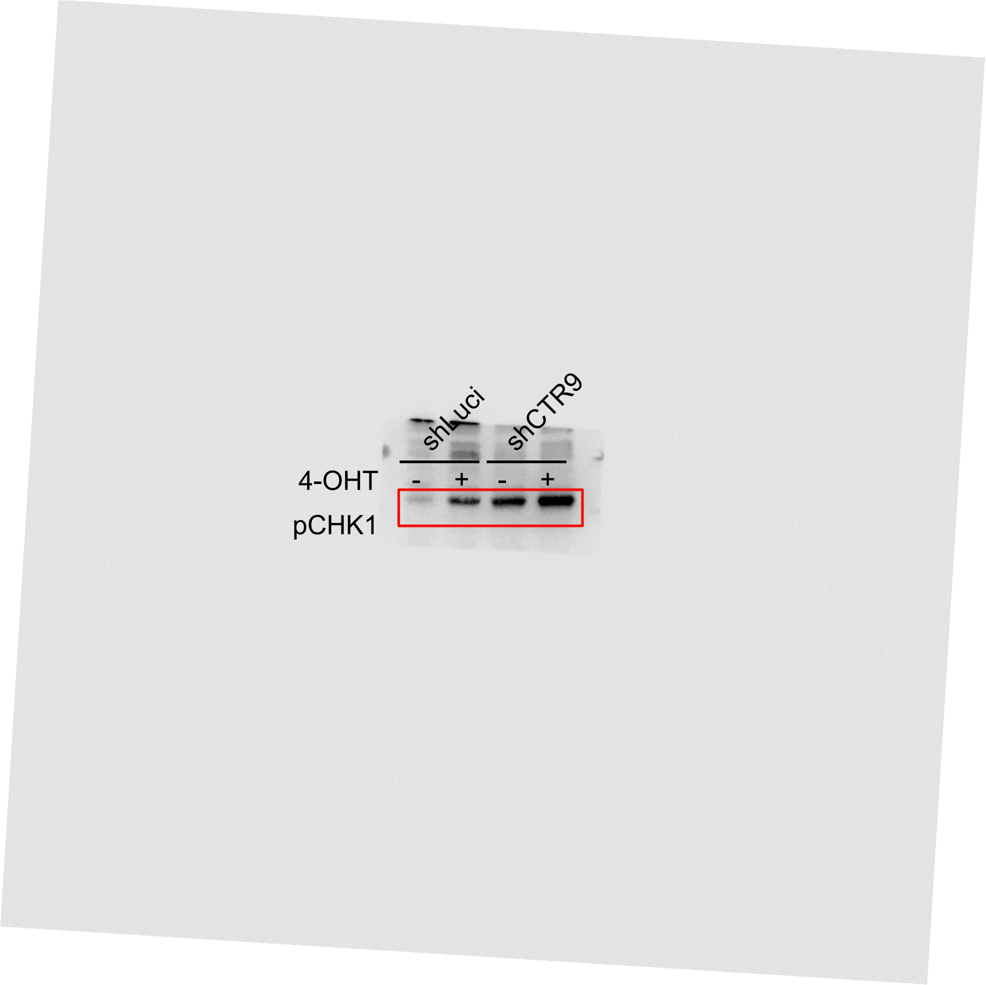

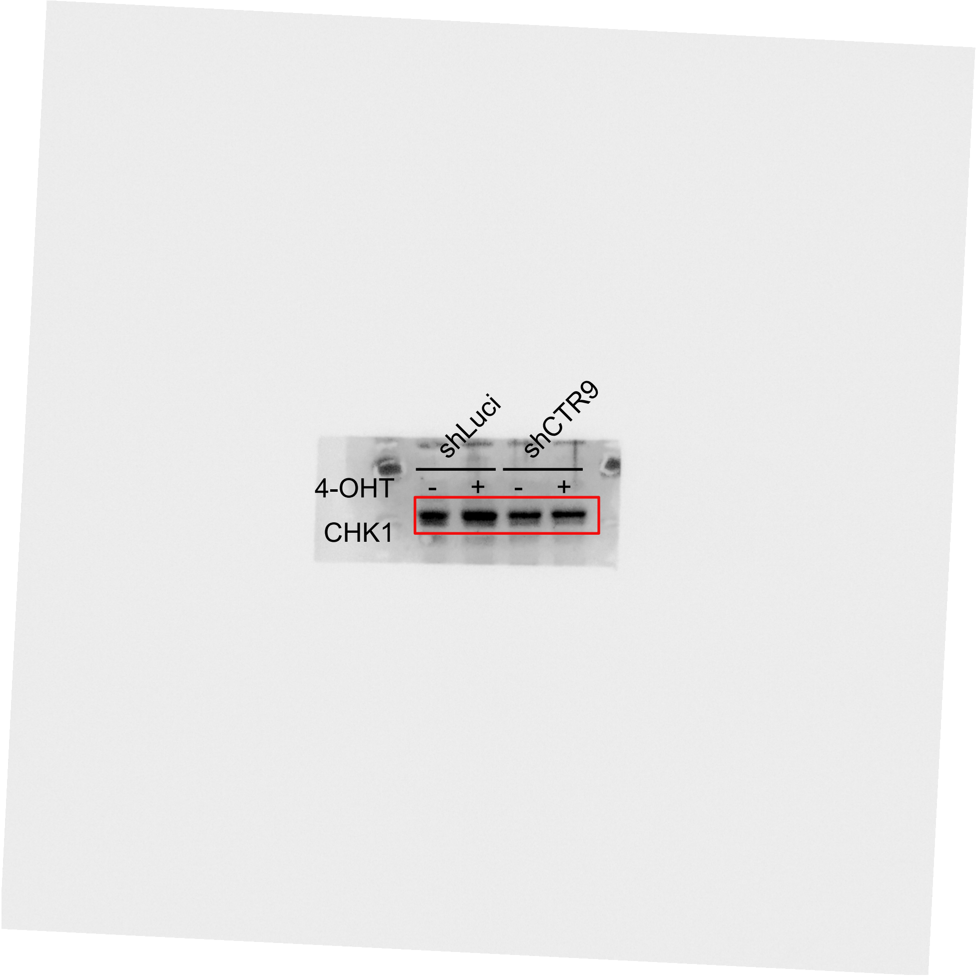

Supplementary Figure 3 panel b.

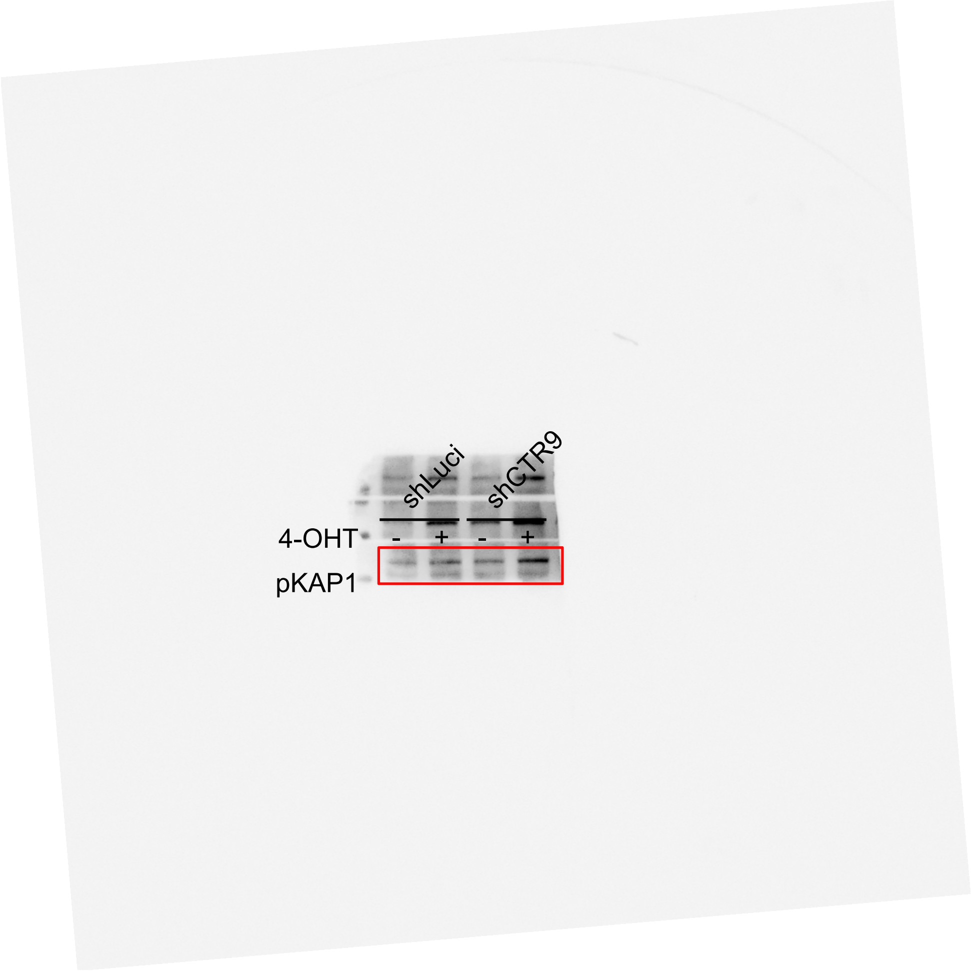

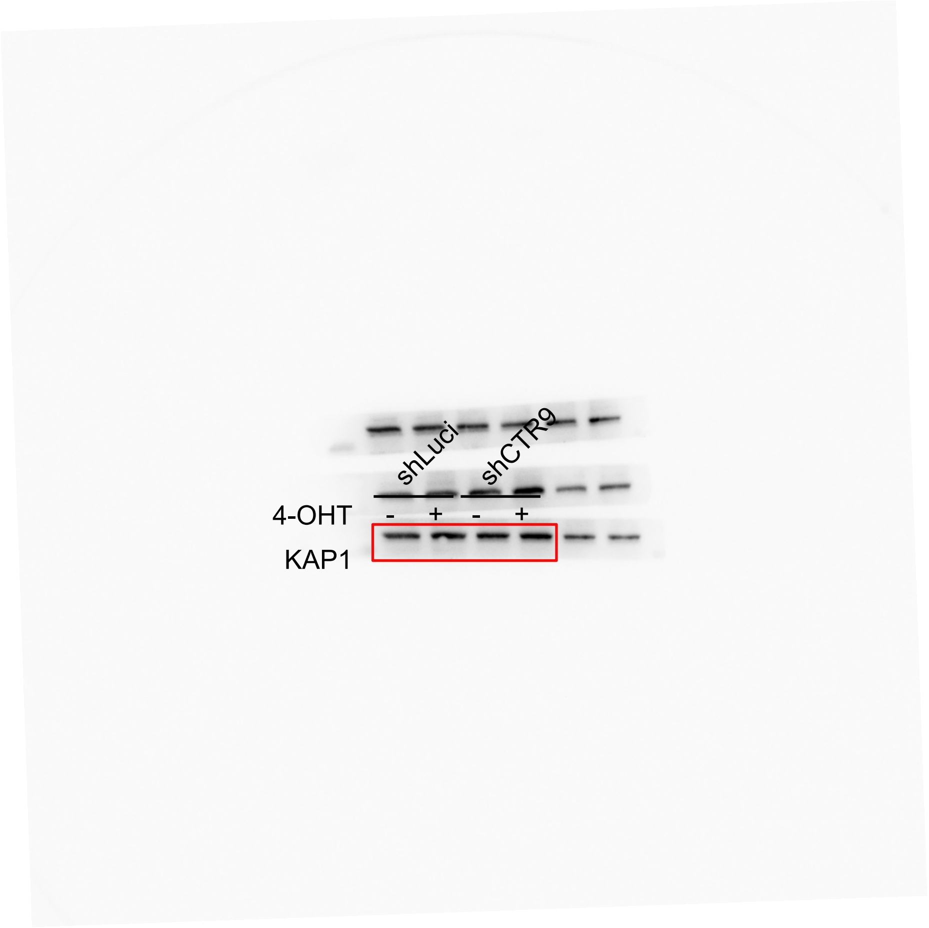

Supplementary Figure 3 panel b.

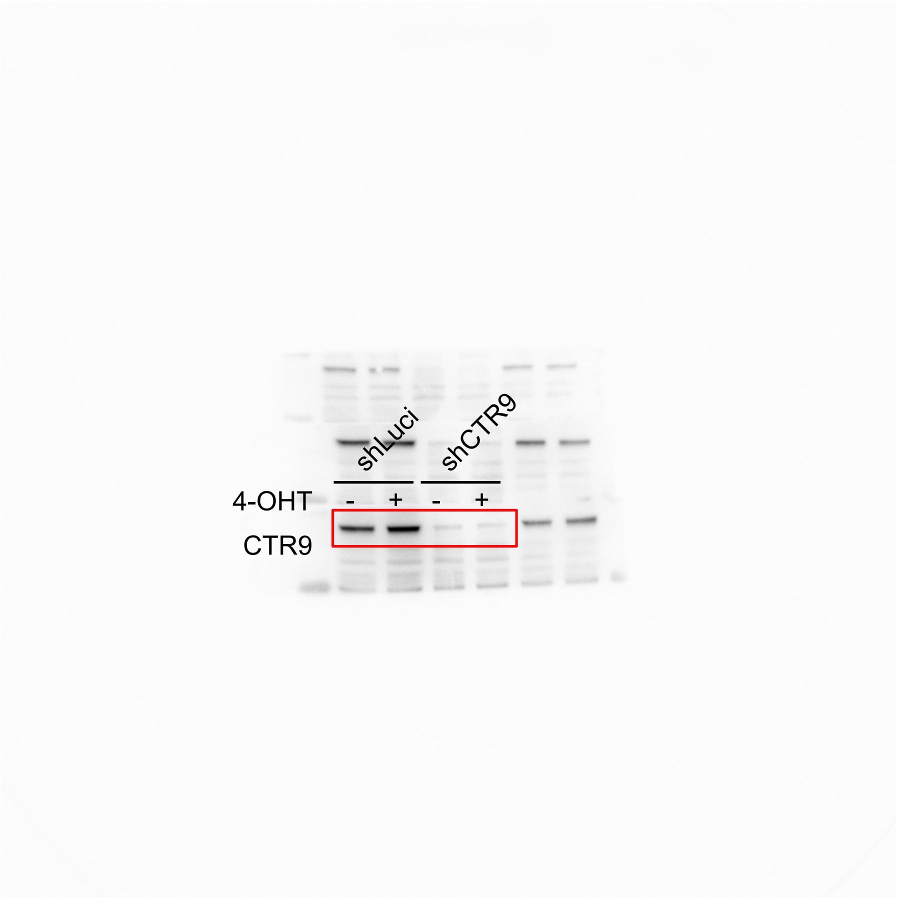

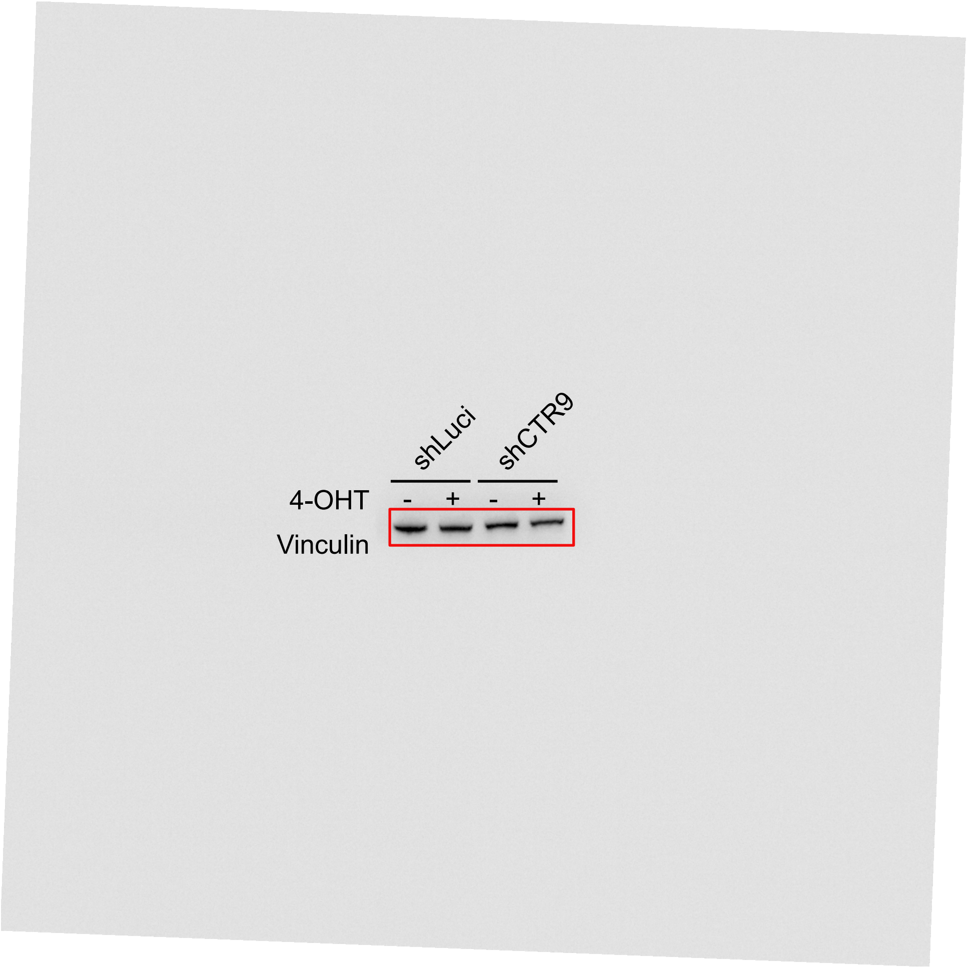

Supplementary Figure 4 panel a.

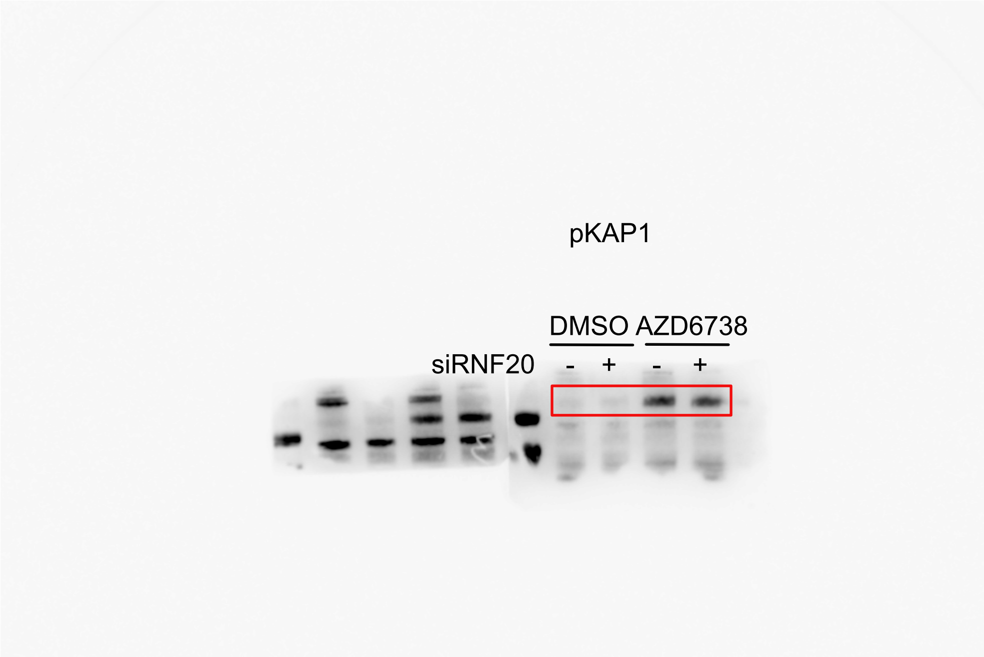

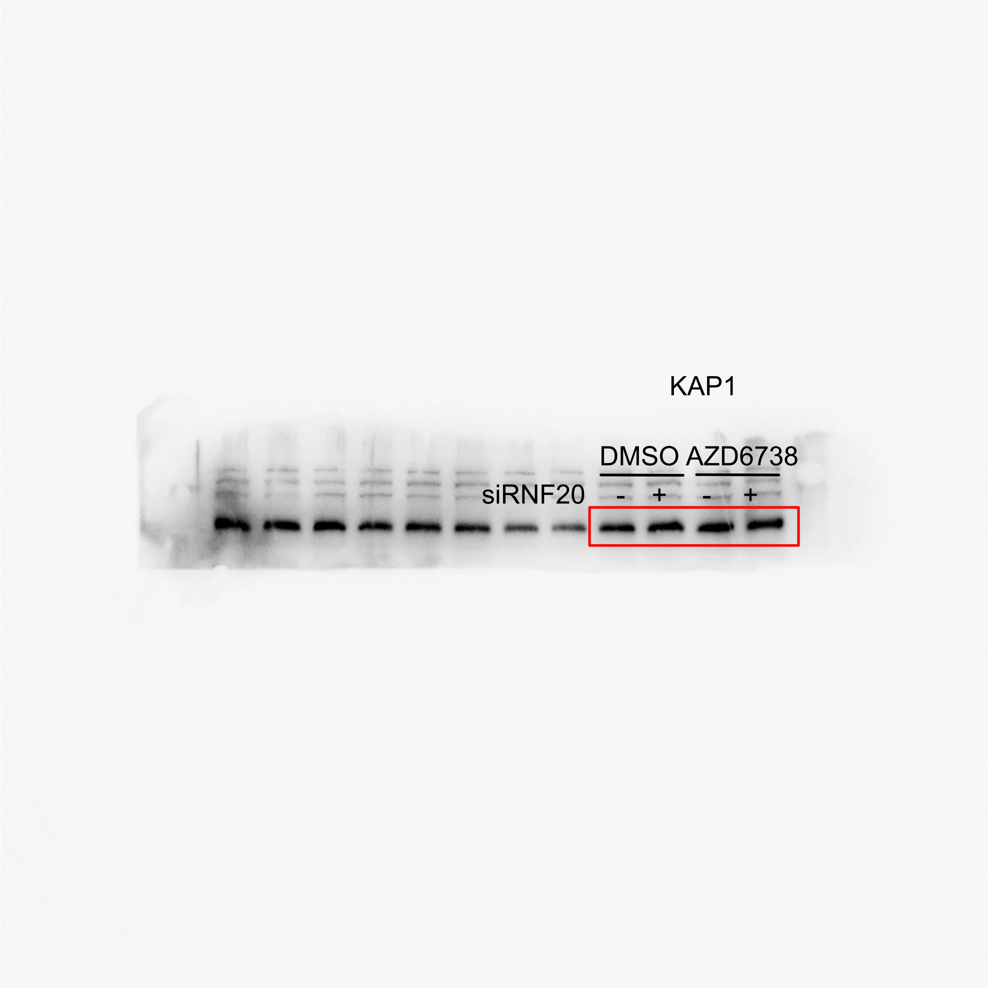

Supplementary Figure 4 panel a.

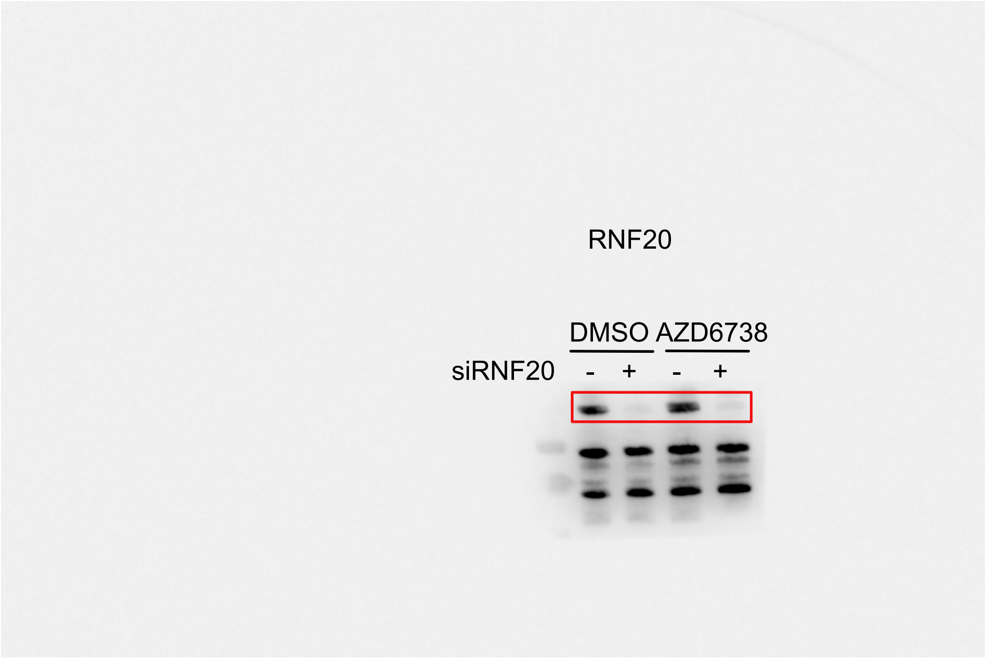

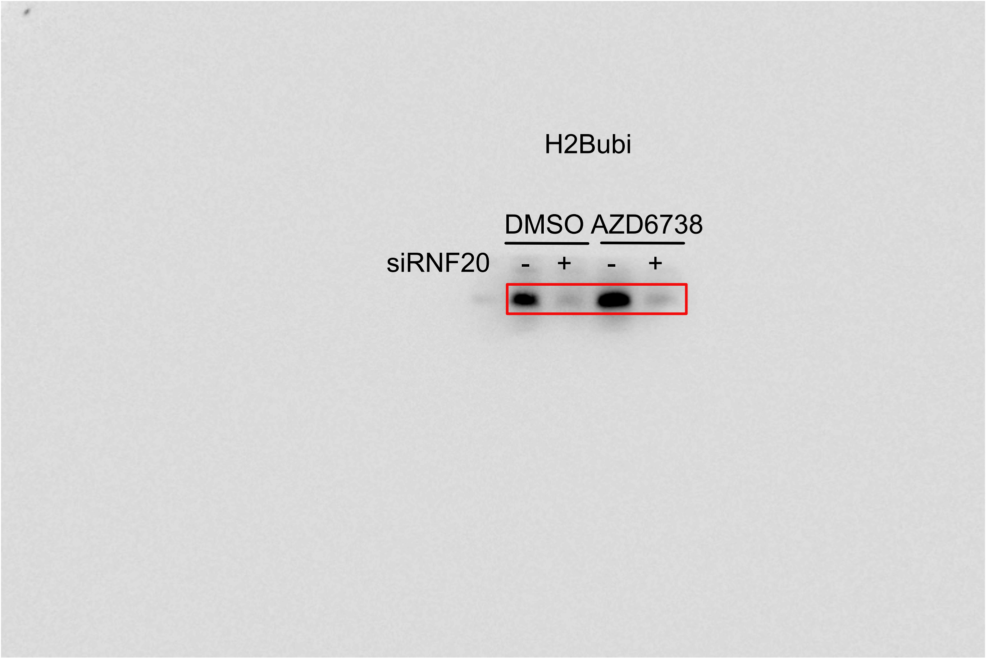

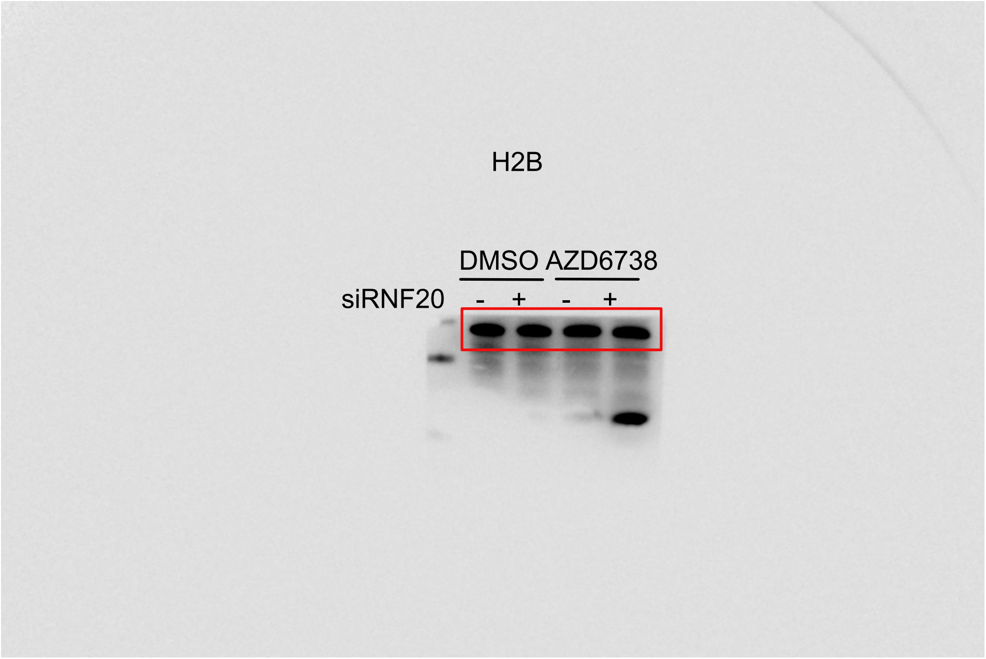

Supplementary Figure 4 panel a.

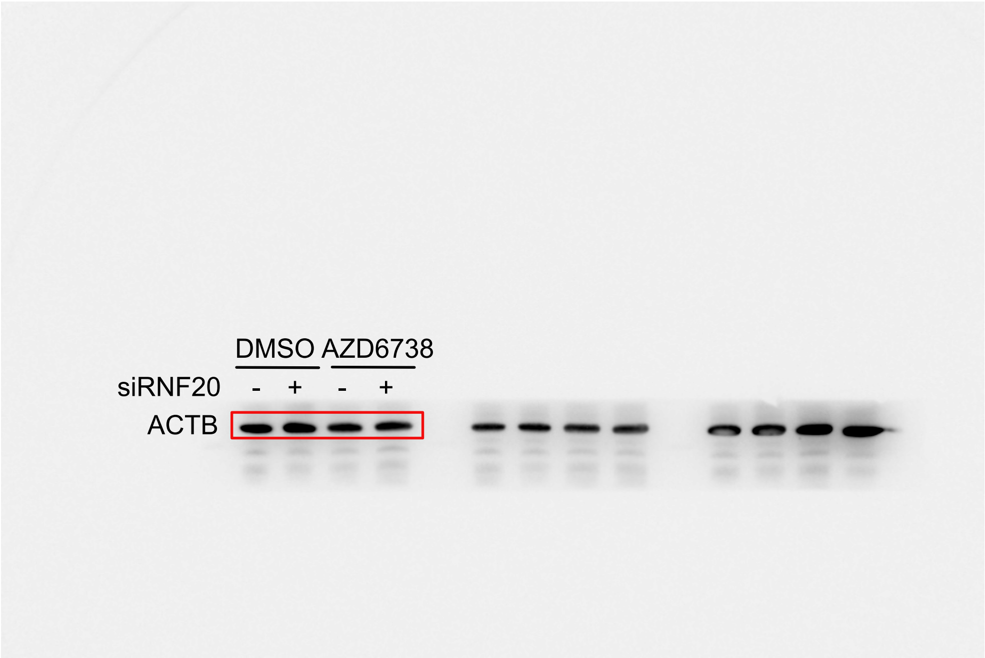

Supplementary Figure 4 panel b.

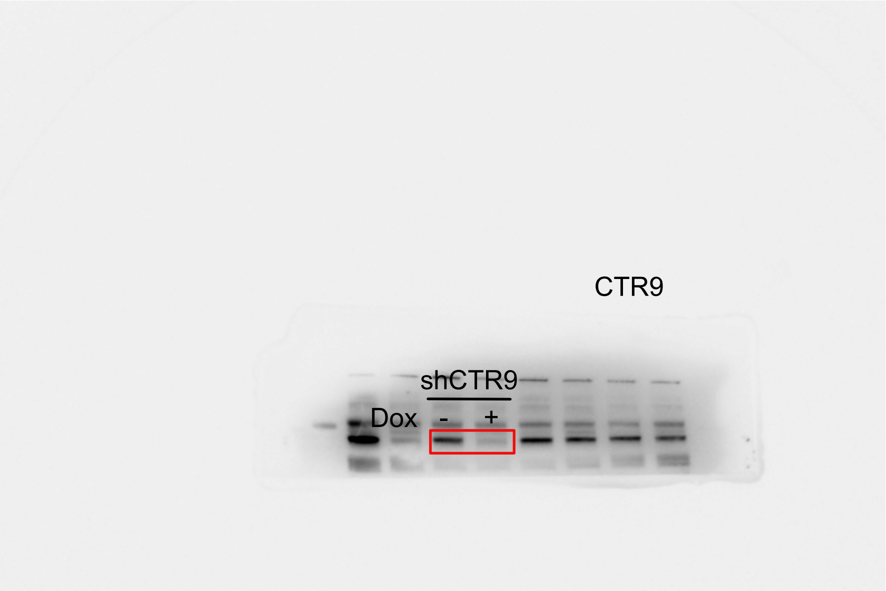

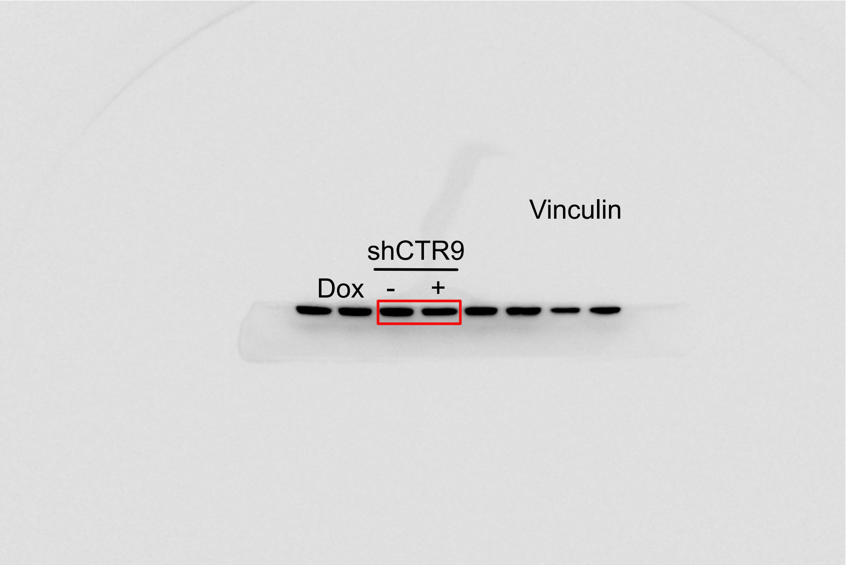

Supplementary Figure 4 panel d.

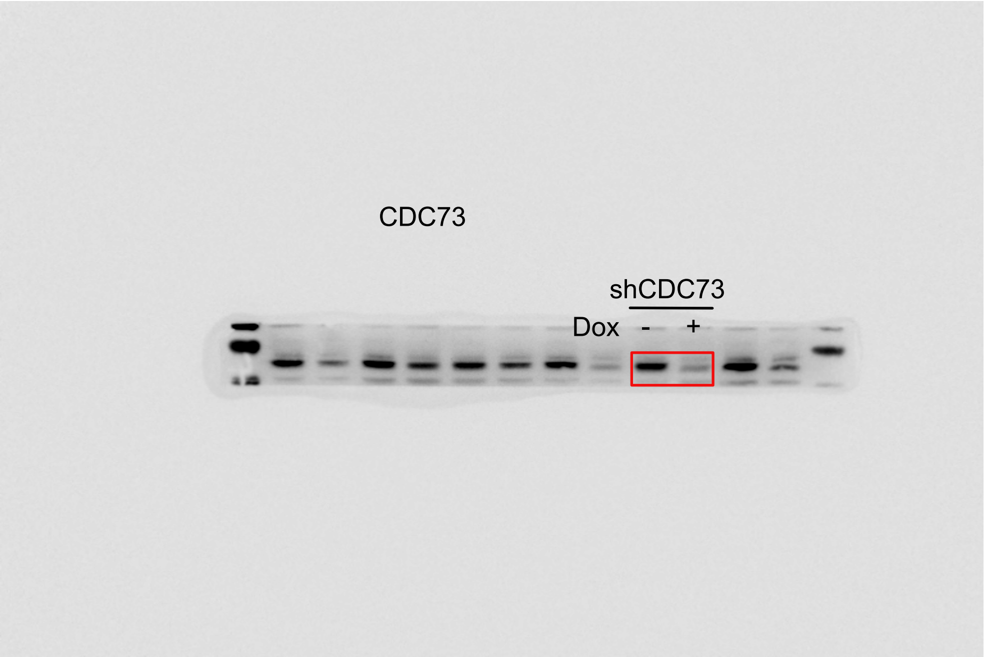

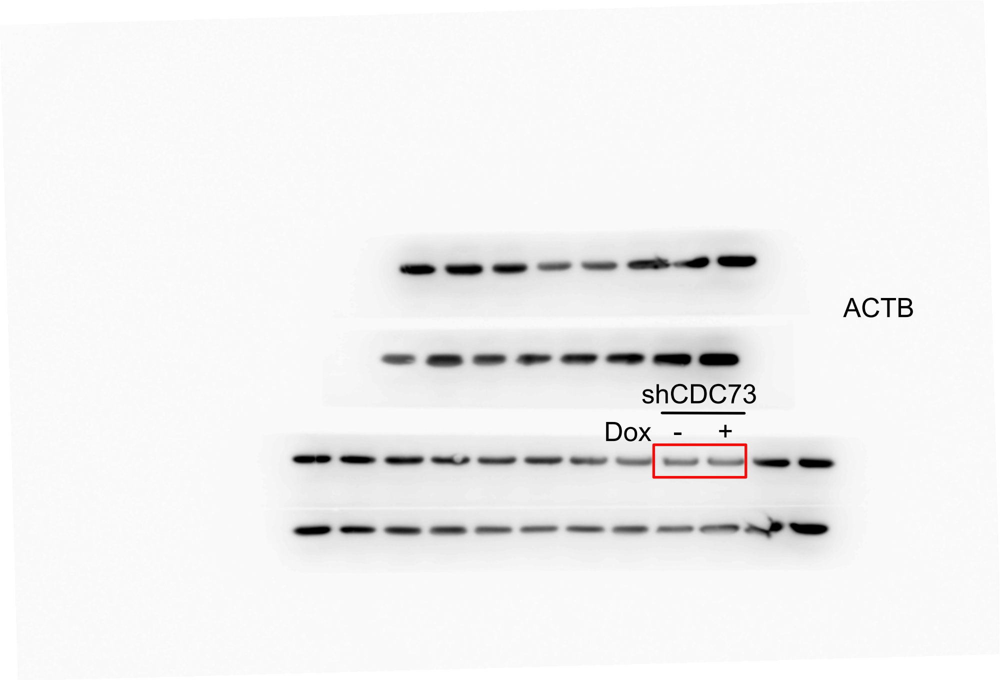

Supplementary Figure 5 panel c.

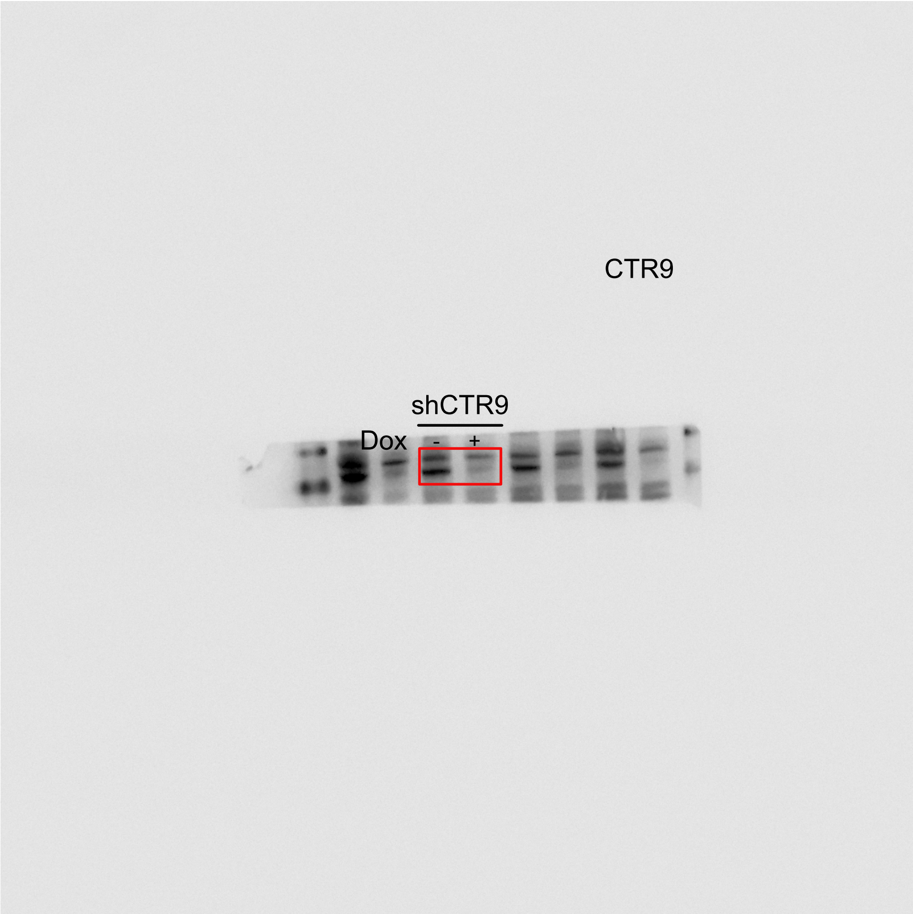

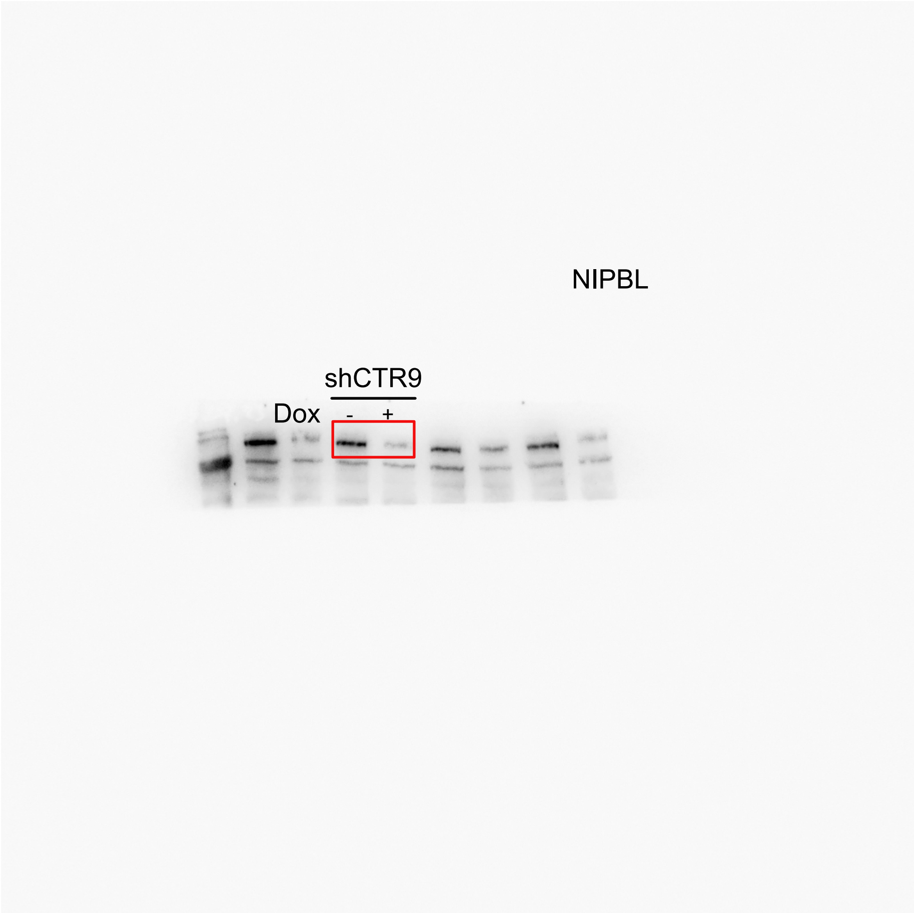

Supplementary Figure 5 panel c.

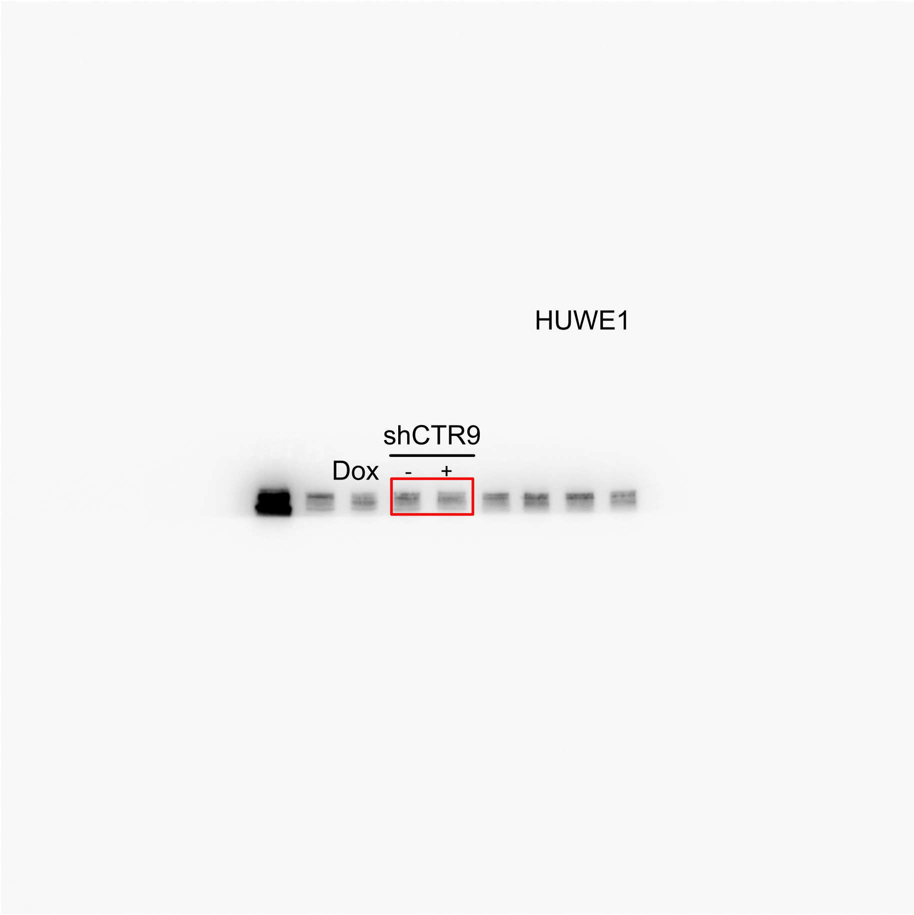

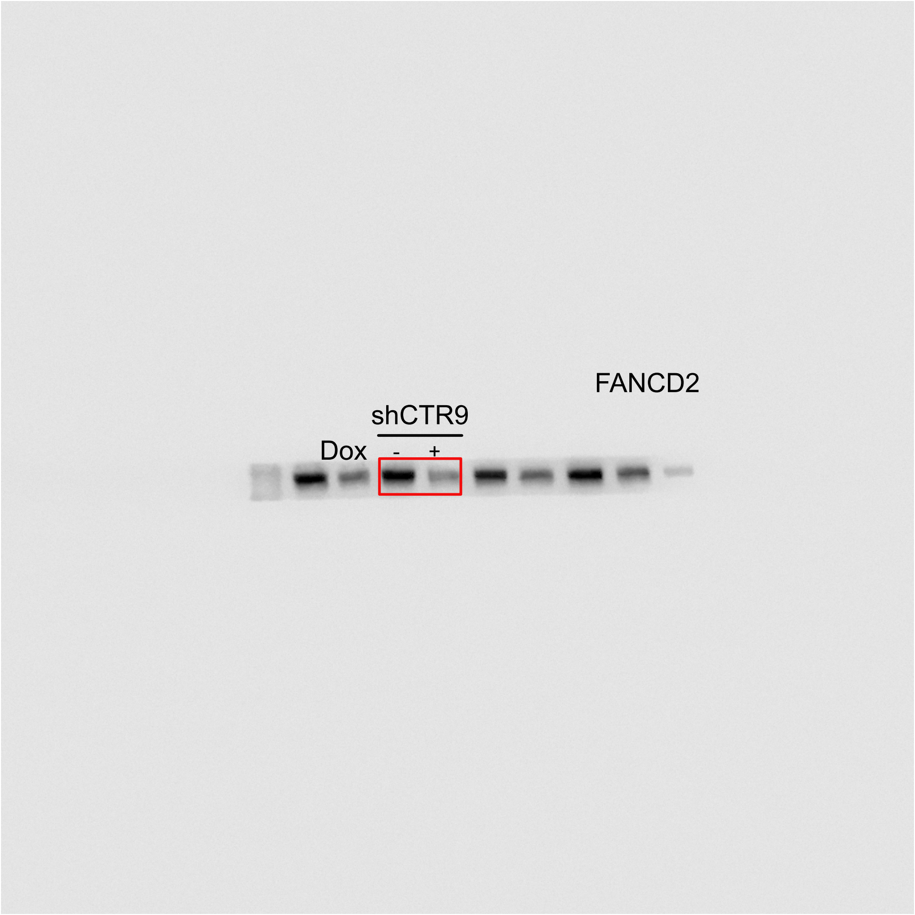

Supplementary Figure 5 panel c.

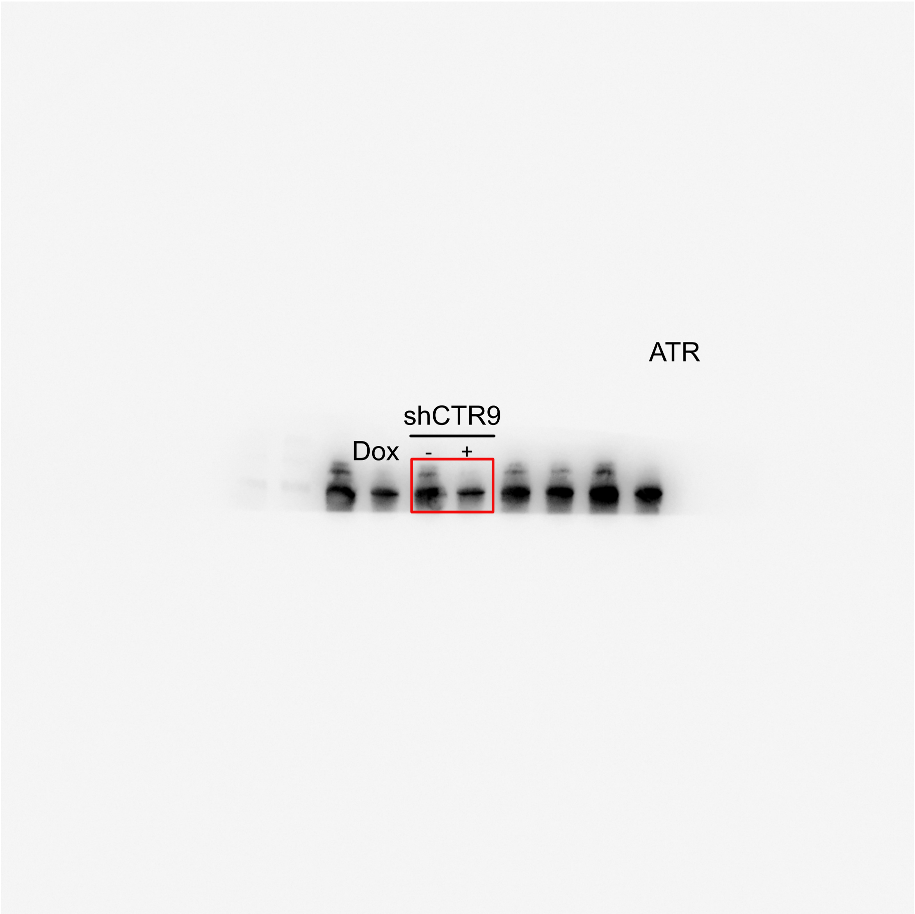

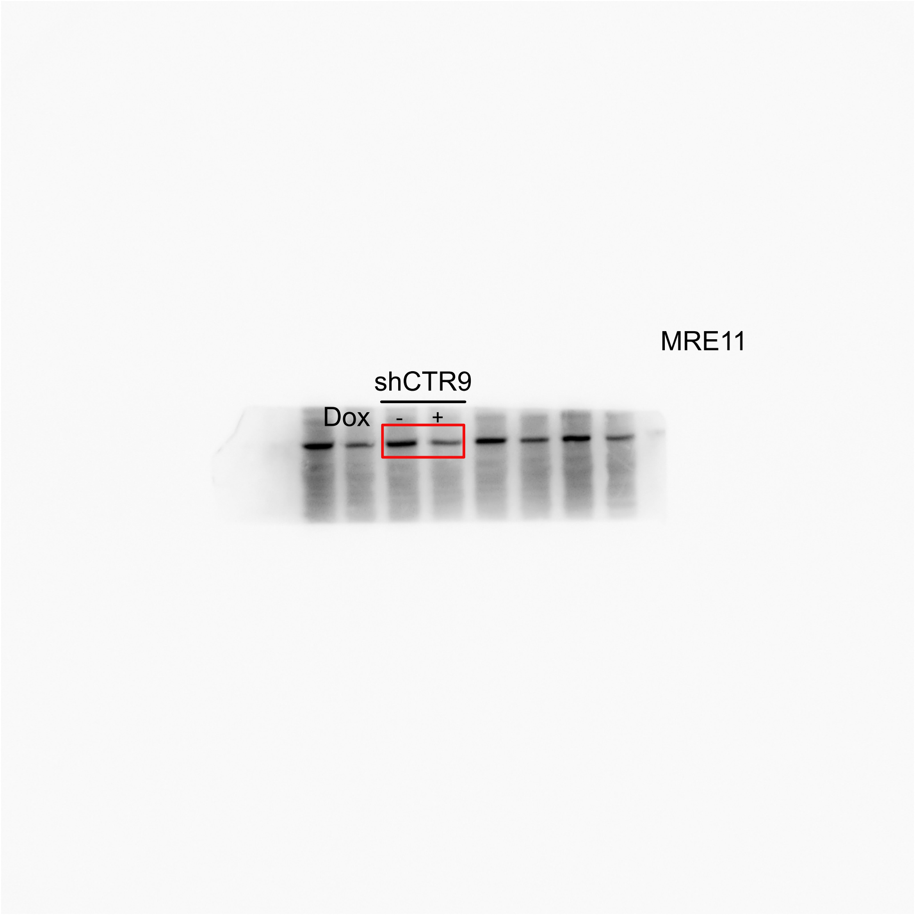

Supplementary Figure 5 panel c.

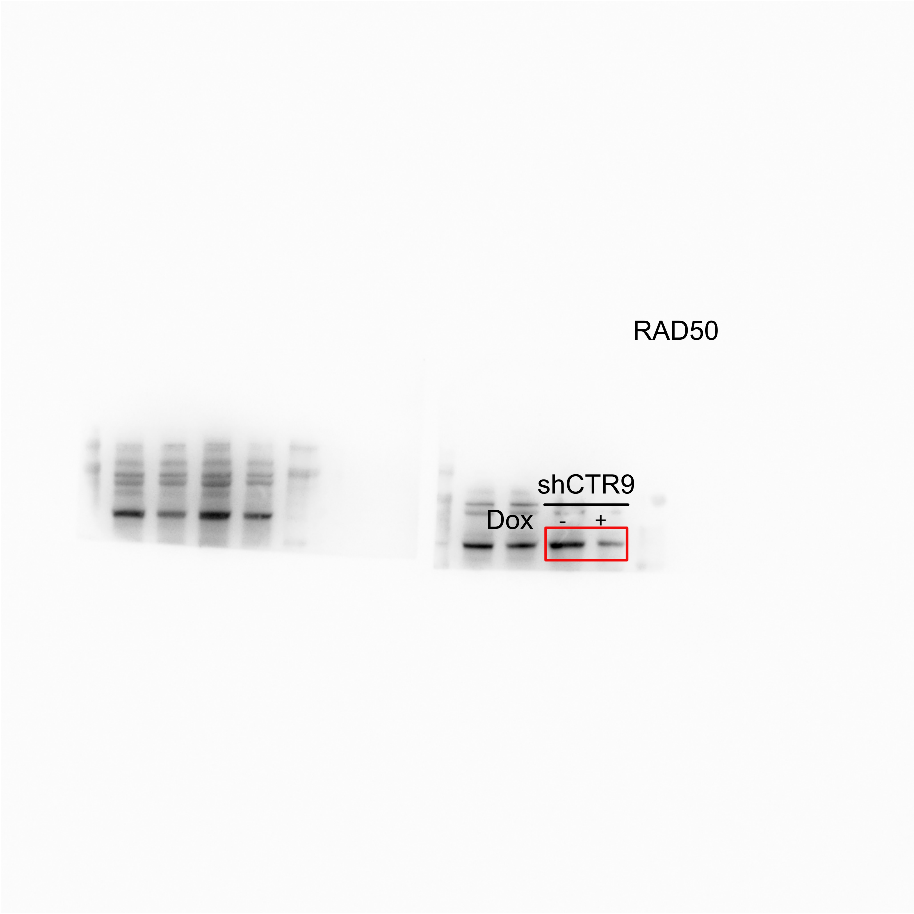

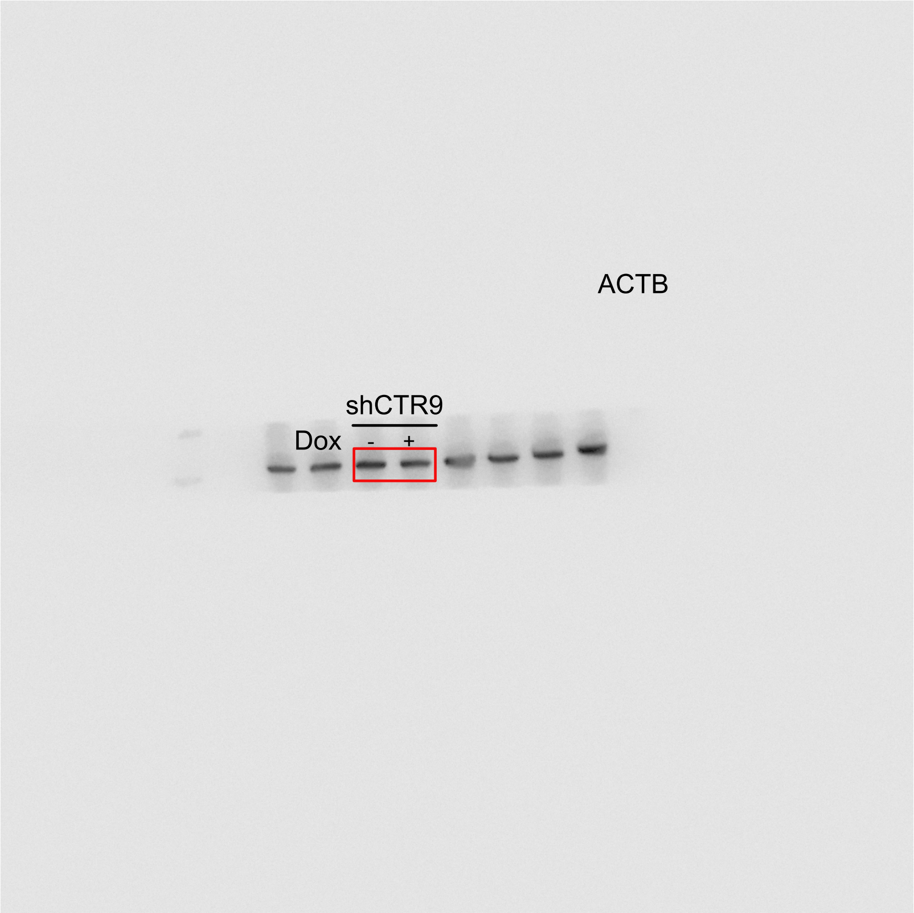

Supplementary Figure 6 panel a.

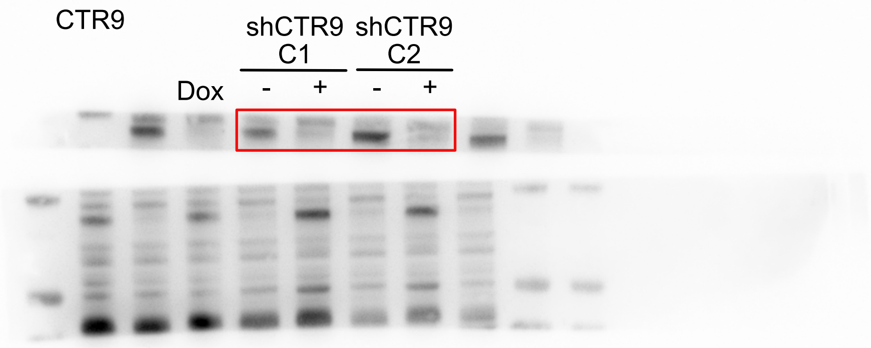

Supplementary Figure 6 panel a.

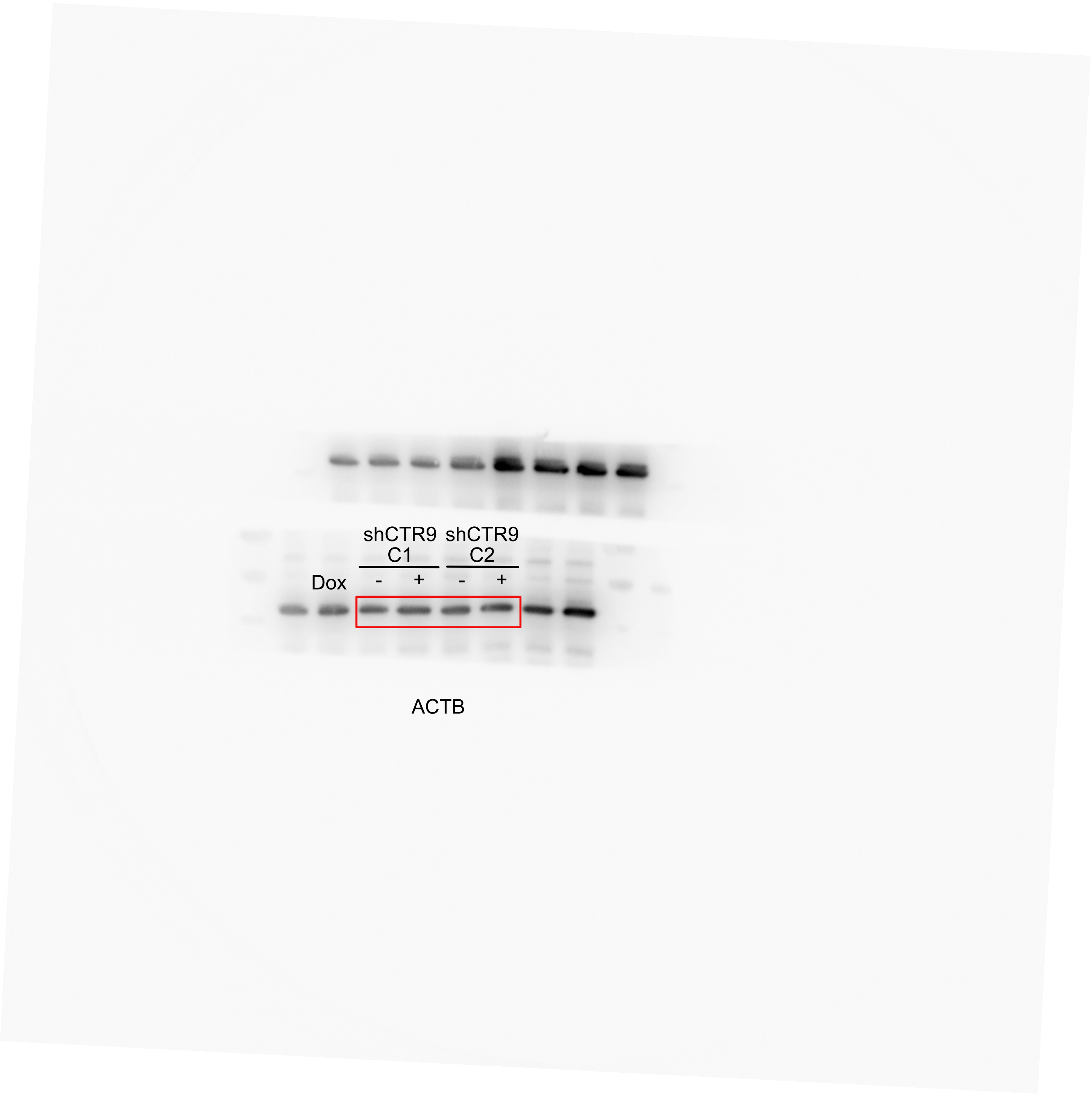

Supplementary Figure 6 panel e.

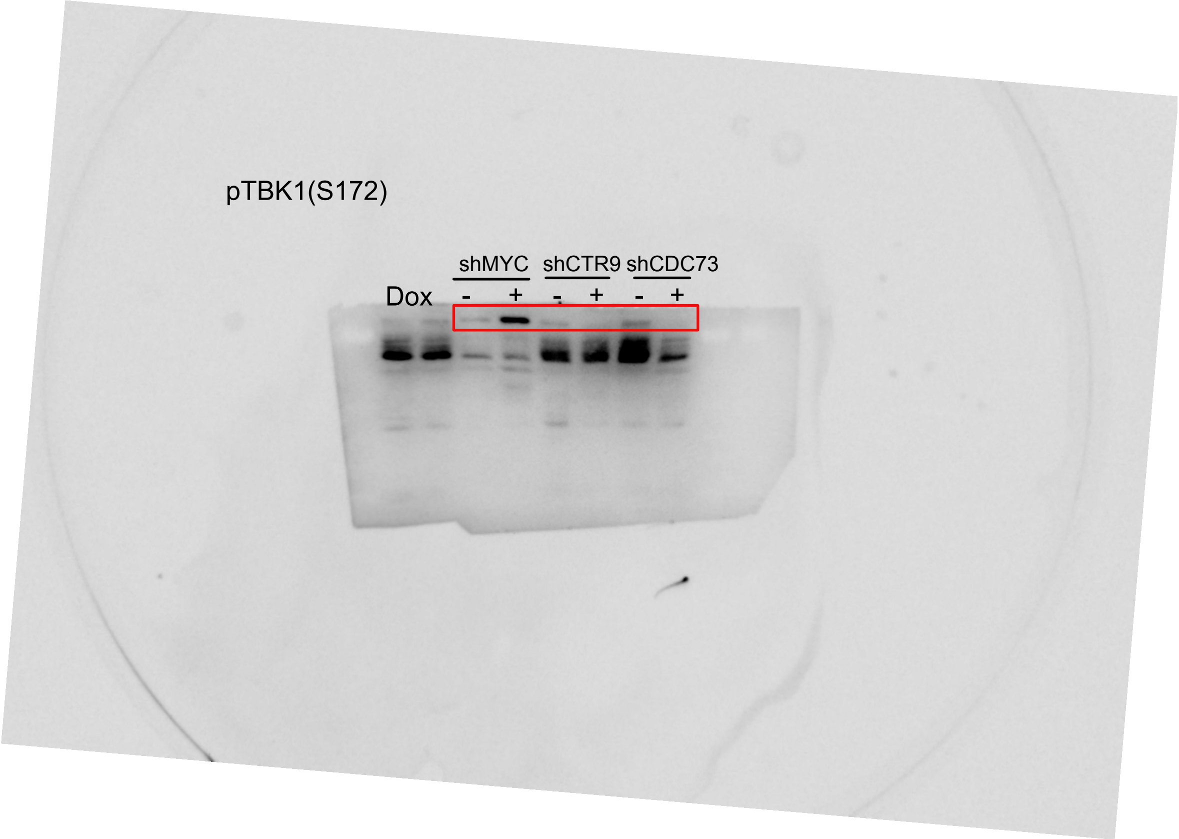

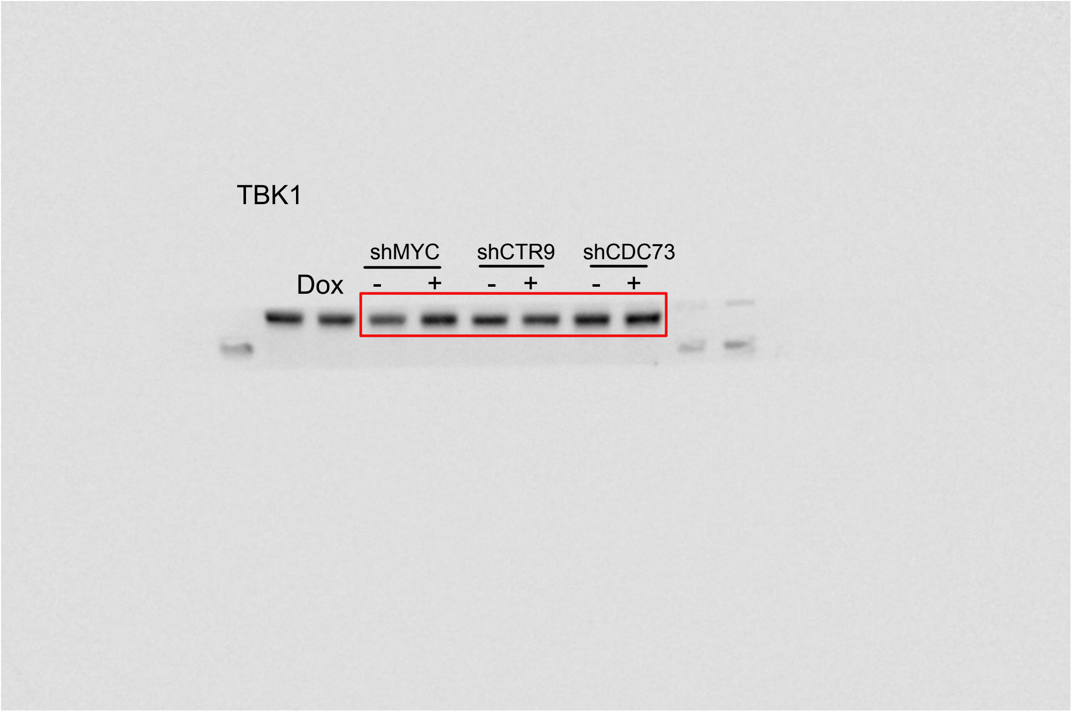

Supplementary Figure 6 panel e.

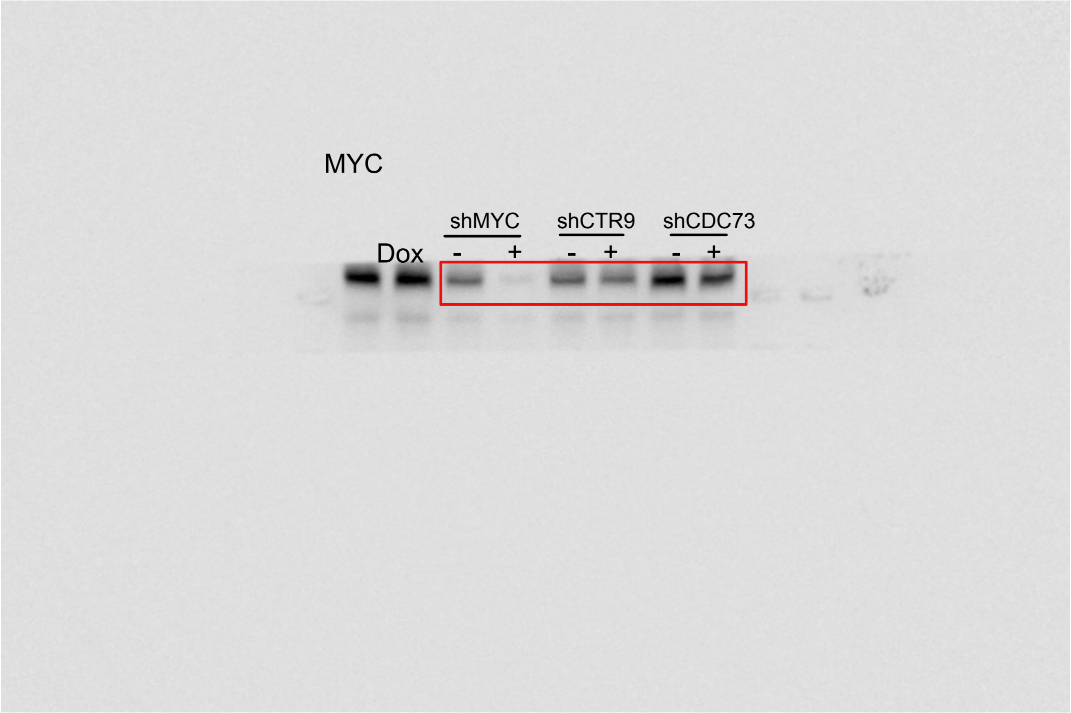

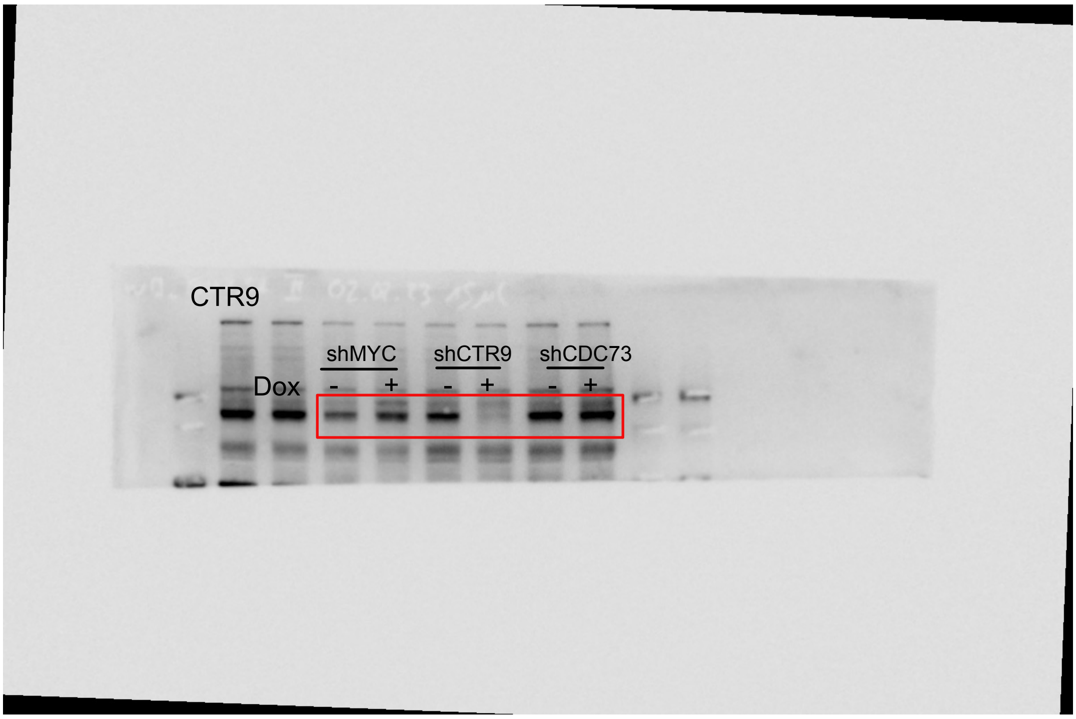

Supplementary Figure 6 panel e.

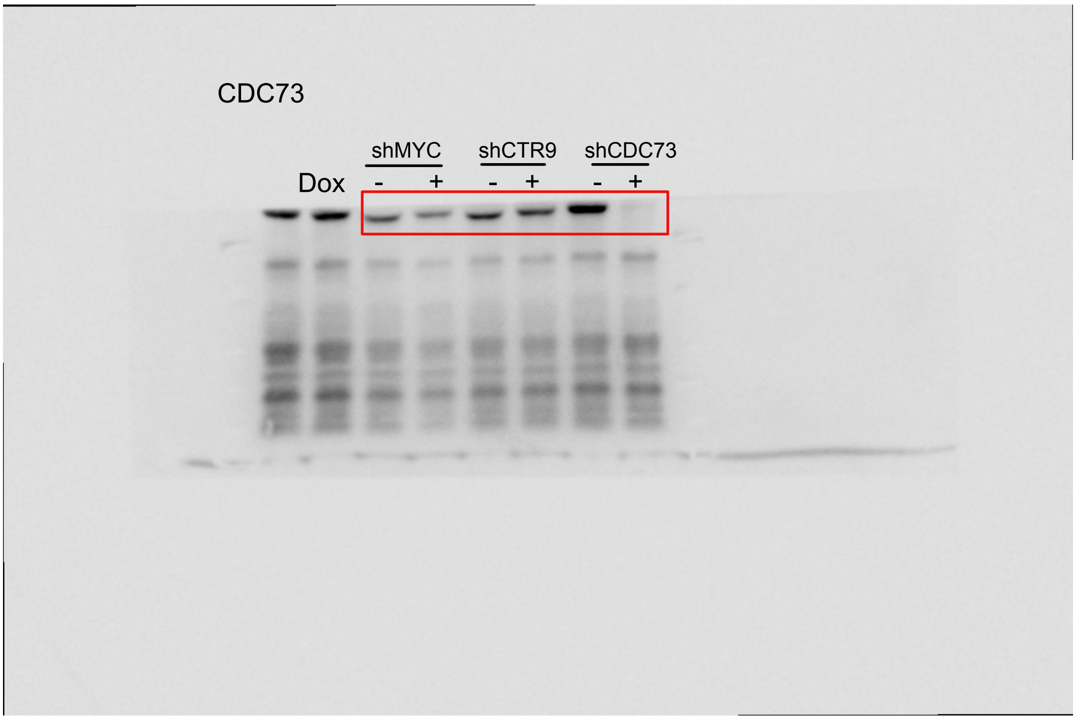

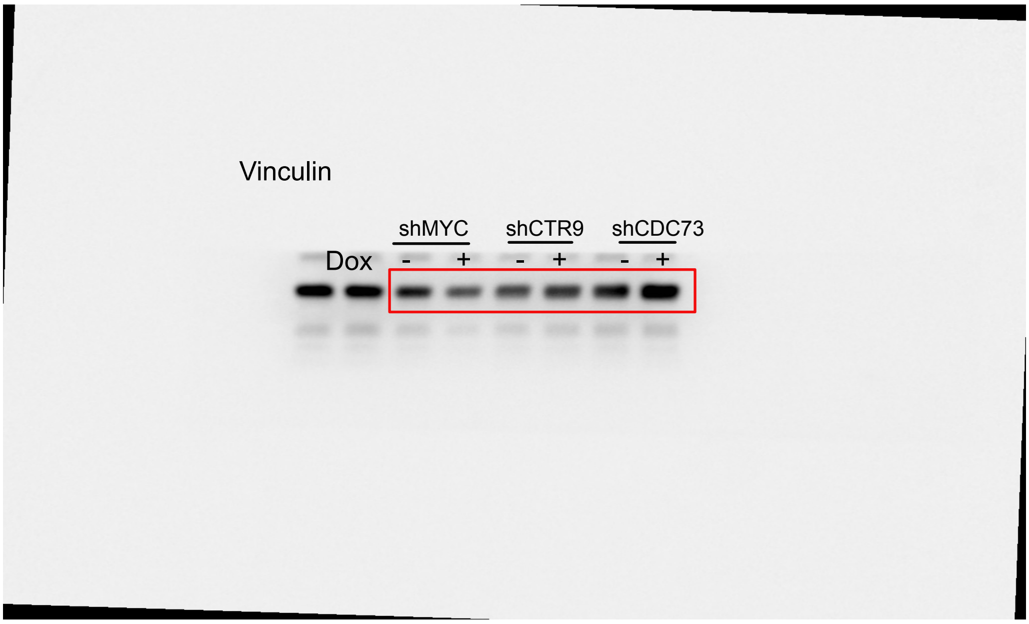

Supplement: Supplementary file 1 — Supplementary Information [file 41467_2024_45760_MOESM1_ESM.pdf]
