# Supplementary material for: PAF1c links S-phase progression to immune evasion and MYC function in pancreatic carcinoma
Source: Nat Commun. 2024 Feb 16;15:1446. doi: 10.1038/s41467-024-45760-8 (PMC10873513; doi:10.1038/s41467-024-45760-8)
Supplement: Supplementary file 3 — Description of Additional Supplementary Files [file 41467_2024_45760_MOESM3_ESM.pdf]

Supplementary Data Legends

Supplementary Data 1: Results of the siRNA screens.

The tables show the individual results for all siRNAs in the three assays shown in Figures 2a and 2b.

Supplementary Data 2: Summary of compound profiling data.

The table shows the percentage of pKAP1-positive cells for all compounds shown in Figure 2e. It also shows the supplier of each compound.

Supplementary Data 3: Primer sequences used in qPCR and ChIP experiments.

The table shows the sequence of the primers used in all experiments.
