# Supplementary material for: PAF1c links S-phase progression to immune evasion and MYC function in pancreatic carcinoma
Source: Nat Commun. 2024 Feb 16;15:1446. doi: 10.1038/s41467-024-45760-8 (PMC10873513; doi:10.1038/s41467-024-45760-8)
Supplement: Supplementary file 7 — Reporting Summary [file 41467_2024_45760_MOESM7_ESM.pdf]

nature portfolio

Corresponding author(s): Martin Eilers

Last updated by author(s): Jan 4, 2024

Reporting Summary

Nature Portfolio wishes to improve the reproducibility of the work that we publish. This form provides structure for consistency and transparency in reporting. For further information on Nature Portfolio policies, see our Editorial Policies and the Editorial Policy Checklist.

Statistics

For all statistical analyses, confirm that the following items are present in the figure legend, table legend, main text, or Methods section.

n/a	Confirmed	
☐	☒	The exact sample size (*n*) for each experimental group/condition, given as a discrete number and unit of measurement
☐	☒	A statement on whether measurements were taken from distinct samples or whether the same sample was measured repeatedly
☐	☒	The statistical test(s) used AND whether they are one- or two-sided<br>*Only common tests should be described solely by name; describe more complex techniques in the Methods section.*
☒	☐	A description of all covariates tested
☐	☒	A description of any assumptions or corrections, such as tests of normality and adjustment for multiple comparisons
☐	☒	A full description of the statistical parameters including central tendency (e.g. means) or other basic estimates (e.g. regression coefficient) AND variation (e.g. standard deviation) or associated estimates of uncertainty (e.g. confidence intervals)
☐	☒	For null hypothesis testing, the test statistic (e.g. *F*, *t*, *r*) with confidence intervals, effect sizes, degrees of freedom and *P* value noted<br>*Give P values as exact values whenever suitable.*
☒	☐	For Bayesian analysis, information on the choice of priors and Markov chain Monte Carlo settings
☒	☐	For hierarchical and complex designs, identification of the appropriate level for tests and full reporting of outcomes
☐	☒	Estimates of effect sizes (e.g. Cohen's *d*, Pearson's *r*), indicating how they were calculated

Our web collection on statistics for biologists contains articles on many of the points above.

Software and code

Policy information about availability of computer code

Data collection

FASTQ Generation software v1.0.0 Illumina https://www.illumina.com/
Operetta High-Content imaging system v4.6
BD FACSDIVATM Software v6.1.2

Data analysis

FastQC v0.11.3 https://www.bioinformatics.babraham.ac.uk/projects/fastqc/
Bowtie2 v2.3.4.1 Langmead and Salzberg, 2012 http://bowtie-bio.sourceforge.net/bowtie2/index.shtml
SAMtools v1.9 Li et al., 2009 http://www.htslib.org/
Integrated Genome Browser v9.1.10 Freese et al., 2016 https://bioviz.org/
GraphPad Prism 9.5.1 for Mac GraphPad software https://www.graphpad.com/scientific-software/prism/
Harmony High Content Imaging and Analysis Software PerkinElmer http://www.perkinelmer.de/product/harmony-4-8-office-hh17000001
StepOne software v2.3 StepOne https://www.thermofisher.com/de/de/home/technical-resources/software-downloads/StepOne-and-StepOnePlus-Real-Time-PCR-System.html
enrichR v3.1 Xie et al., 2021 https://cran.r-project.org/web/packages/enrichR/vignettes/enrichR.html
GOstats v2.64.0 Falcon et al., 2007 https://bioconductor.org/packages/release/bioc/html/GOstats.html
Rsubread v2.12.2 Liao et al., 2019 https://bioconductor.org/packages/release/bioc/html/Rsubread.html
R version v4.2.2 The R Foundation https://www.R-project.org/
DeepTools v3.5.1 Ramirez et al., 2016 https://deeptools.readthedocs.io/en/develop/
ChIPseeker v1.34.1 Wang et al., 2022 https://guangchuangyu.github.io/software/ChIPseeker
UMI-tools v1.0.1 Smith et al., 2017 https://umi-tools.readthedocs.io/en/latest/
Living Image® Software - PerkinElmer® IVIS® Systems, https://www.perkinelmer.com/in_vivo/imaging

QuPath v0.3.2 Bankhead et al., 2017 https://qupath.github.io/<br>FlowJo software (Treestar, version 10.9)

For manuscripts utilizing custom algorithms or software that are central to the research but not yet described in published literature, software must be made available to editors and reviewers. We strongly encourage code deposition in a community repository (e.g. GitHub). See the Nature Portfolio guidelines for submitting code & software for further information.

Data

Policy information about availability of data

All manuscripts must include a data availability statement. This statement should provide the following information, where applicable:

- Accession codes, unique identifiers, or web links for publicly available datasets
- A description of any restrictions on data availability
- For clinical datasets or third party data, please ensure that the statement adheres to our policy

ChIP-Rx, BLISS and RNA sequencing data are available from the Gene Expression Omnibus under accession number GSE228800, including GSE228795 and GSE243703 (ChIP-seq/CUT&RUN), GSE228797 (RNA-seq), GSE228799 (BLISS) and GSE243701 (4sU-seq). The mm10 (GRCm38) and hg19 (GRCh37.p13) reference genomes are available at https://www.ncbi.nlm.nih.gov/datasets/genome/GCF_000001635.20/ and https://www.ncbi.nlm.nih.gov/datasets/genome/GCF_000001405.25/, respectively. The mm10 blacklisted regions are available at https://www.encodeproject.org/files/ENCFF543DDX/. Source data are provided with this paper.

Research involving human participants, their data, or biological material

Policy information about studies with human participants or human data. See also policy information about sex, gender (identity/presentation), and sexual orientation and race, ethnicity and racism.

Reporting on sex and gender	N/A
Reporting on race, ethnicity, or other socially relevant groupings	N/A
Population characteristics	N/A
Recruitment	N/A
Ethics oversight	N/A

Note that full information on the approval of the study protocol must also be provided in the manuscript.

Field-specific reporting

Please select the one below that is the best fit for your research. If you are not sure, read the appropriate sections before making your selection.

☒ Life sciences ☐ Behavioural & social sciences ☐ Ecological, evolutionary & environmental sciences

For a reference copy of the document with all sections, see nature.com/documents/nr-reporting-summary-flat.pdf

Life sciences study design

All studies must disclose on these points even when the disclosure is negative.

Sample size	1) Sample sizes were not predetermined and were chosen based on experience and common practice in the field. Microscopy experiments sampled at least 500 cells per condition. For other in vitro experiments, sample sizes ranged from several thousand to millions of cells, depending on the type of experiment.<br>2) Animal experiments were performed with at least 3 animals per condition.
Data exclusions	No data was excluded from the analysis.
Replication	Most experiments were done in biological triplicates. For each experiment we state the number of independent replications in the figure legend.
Randomization	Experiments performed with cell lines are naturally randomized.<br>Group division in animal experiments was randomnized by sorting by strength of luciferase signal, to ensure each group has the same starting point.
Blinding	Blinding was not applied since it is not feasible for many of the applications used in our manuscript.

Reporting for specific materials, systems and methods

We require information from authors about some types of materials, experimental systems and methods used in many studies. Here, indicate whether each material, system or method listed is relevant to your study. If you are not sure if a list item applies to your research, read the appropriate section before selecting a response.

Materials & experimental systems

n/a	Involved in the study
☐	☒ Antibodies
☐	☒ Eukaryotic cell lines
☒	☐ Palaeontology and archaeology
☐	☒ Animals and other organisms
☒	☐ Clinical data
☒	☐ Dual use research of concern
☒	☐ Plants

Methods

n/a	Involved in the study
☐	☒ ChIP-seq
☐	☒ Flow cytometry
☒	☐ MRI-based neuroimaging

Antibodies

Antibodies used

CD3 Abcam ab16669 1:100 (IHC)
CD4 Thermo Fisher Scientific 14-9766-82 1:100 (IHC)
CD8 Lab Vision RB-9009-P1 1:100 (IHC)
F4/80 Abcam ab6640 1:1000 (IHC)
CD45R/B220 BD Biosciences 550286 1:50 (IHC)
KAP1 phospho-S824 Abcam ab70369 1:500 (IHC), 1:5000 (WB), 1:600 (IF)
gamma H2AX Abcam ab2893 1:1000 (IHC)
MYC Abcam ab32072 1:5000 (WB)
CTR9 Bethyl A301-395A 1:1000 (WB), 15 µg (ChIP-seq.)
CDC73 Cell Signaling Technology 3644 1:1000 (WB)
RTF1 Bethyl A300-179A 1:1000 (WB)
Vinculin Sigma-Aldrich V9131 1:5000 (WB)
Actin-beta Sigma-Aldrich A5441 1:5000 (WB)
KAP1 Bethyl A300-274A 1:1000 (WB)
TBK1 phospho-S172 Cell Signaling Technology 5483 1:1000 (WB)
TBK1 Cell Signaling Technology 3504 1:1000 (WB)
Total RNAPII (F-12) Santa Cruz Biotechnology sc-55492 1:1500 (PLA)
gamma H2AX Cell Signaling Technology 2577 1:500 (IF)
PCNA Abcam ab92552 1:1500 (PLA)
RAD9 Thermo Fisher Scientific PA5-21275 (PLA)
RNAPII phospho Ser5 BioLegend 904001 1:500 (PLA), 3 µg (ChIP)
CTR9 Novus NB100-68205 1:500 (PLA)
Total RNAPII (A-10) Santa Cruz Biotechnology sc-17798 15 µg (ChIP-seq.), 3 µg (ChIP)
SPT6 Novus NB100-2582 10 µg (ChIP-seq.), 1.5 µg (ChIP)
SPT5 Santa Cruz Biotechnology sc-133217 15 µg (ChIP-seq.), 3 µg (ChIP)
RNAPII phosphor-Ser2 Abcam ab5095 3 µg (ChIP)
SPT4 Cell Signaling Technology 64828 1.5 µg (ChIP)
NIPBL Santa Cruz Biotechnology sc-374625 1:1000 (WB)
MRE11 Abcam ab208020 1:1000 (WB)
CHK1 (phosphoSer345) Cell Signaling Technology 2348 (WB)
RAD50 Abcam ab208019 1:1000 (WB)
ATR Thermo Fisher Scientific A300-138A 1:1000 (WB)
CHK1 Santa Cruz Biotechnology sc-7898 1:1000 (WB)
FANCD2 Abcam ab108928 1:1000 (WB)
HUWE1 Abcam ab70161 1:1000 (WB)
RNF20 Cell Signaling Technology 11974 1:1000 (WB)
Ubiquityl-Histone H2B (Lys120) Cell Signaling Technology 5546 1:1000 (WB)
histone H2B Abcam ab1790 1:1000 (WB)
CD45 BioLegend BV510 30-F11 1:300 (Flow Cyt)
CD3e BioLegend AF700 500A2 1:300 (Flow Cyt)
PD-1/CD279 BioLegend BV421 29F.1A12 1:200 (Flow Cyt)
CD11b BioLegend PE M1/70 1:300 (Flow Cyt)
CD11c BioLegend AF647 N418 1:300 (Flow Cyt)
CD4 BioLegend BV650 GK1.5 1:200 (Flow Cyt)
CD4 BioLegend BV605 GK1.5 1:200 (Flow Cyt)
CD8 BioLegend FITC 53-6.7 1:200 (Flow Cyt)
CD8 BioLegend PE 53-6.7 1:200 (Flow Cyt)
CTLA-4 BioLegend PE UC10-4B9 1:200 (Flow Cyt)
CD45R/B220 BioLegend PE-Dazzle RA3-6B2 1:200 (Flow Cyt)
Ly6G BioLegend PerCP-Cy5.5 1A8 1:300 (Flow Cyt)

Ly6C BioLegend AF700 HK1.4 1:300 (Flow Cyt)
anti-F4/80 BioLegend BV421 BM8 1:300 (Flow Cyt)
Podoplanin BioLegend APC 8.1.1 1:300 (Flow Cyt)
Ep-CAM BioLegend BV421 G8.8 1:300 (Flow Cyt)
E-Cadherin BioLegend PE-Dazzle DECMA-1 1:200 (Flow Cyt)
CTLA-4 BioLegend PerCP-Cy5.5 UC10-4B9 1:200 (Flow Cyt)
CD206 BioLegend BV650 C068C2 1:200 (Flow Cyt)
CD31 eBioscience PE-Cy7 390 1:200 (Flow Cyt)
CD44 eBioscience SB645 IM7 1:200 (Flow Cyt)
MHCII SB600 ThermoFisher M5/114.15.2 1:200 (Flow Cyt)
PDGFRa ThermoFisher SB702 APA5 1:200 (Flow Cyt)
H-2Db Thermo Fisher Scientific MA5-17992 1 µg (Flow Cyt)
H-2Kb Bio X Cell BE0172 1 µg (Flow Cyt)
ECL anti-mouse IgG horseradish peroxidase Sigma-Aldrich GENA931-1ML 1:5000 (WB)
ECL anti-rabbit IgG horseradish peroxidase Sigma-Aldrich GENA934-1ML 1:5000 (WB)
Alexa Fluor® 647 Goat anti-rabbit IgG Thermo Fisher Scientific A-21244 1:400 (IF)
Alexa Fluor® 488 Goat anti-rabbit IgG Thermo Fisher Scientific A-11008 1:400 (IF)

Validation

CD3 https://www.abcam.com/products/primary-antibodies/cd3-antibody-sp7-ab16669.html
CD4 https://www.thermofisher.com/antibody/product/CD4-Antibody-clone-4SM95-Monoclonal/14-9766-82
F4/80 https://www.abcam.com/products/primary-antibodies/f480-antibody-cia3-1-macrophage-marker-ab6640.html
CD45R/B220 https://www.bdbiosciences.com/en-ca/products/reagents/flow-cytometry-reagents/research-reagents/single-color-antibodies-ruo/purified-rat-anti-mouse-cd45r.550286
KAP1 phospho-S824 https://www.abcam.com/products/primary-antibodies/kap1-phospho-s824-antibody-ab70369.html
gamma H2AX https://www.abcam.com/products/primary-antibodies/gamma-h2ax-phospho-s139-antibody-ab2893.html
MYC https://www.abcam.com/products/primary-antibodies/c-myc-antibody-y69-chip-grade-ab32072.html
CTR9 https://www.fortislife.com/products/primary-antibodies/rabbit-anti-ctr9-antibody/BETHYL-A301-395
CDC73 https://www.cellsignal.com/products/primary-antibodies/cdc73-a264-antibody/3644
RTF1 https://www.fortislife.com/products/primary-antibodies/rabbit-anti-rtf1-antibody/BETHYL-A300-179
Vinculin https://www.sigmaaldrich.com/DE/en/product/sigma/v9131
Actin-beta https://www.sigmaaldrich.com/DE/en/product/sigma/a5441
KAP1 https://www.biomol.com/products/antibodies/primary-antibodies/general/anti-kap-1-a300-274a-t
TBK1 phospho-S172 https://www.cellsignal.com/products/primary-antibodies/phospho-tbk1-nak-ser172-d52c2-xp-rabbit-mab/5483
TBK1 https://www.cellsignal.com/products/primary-antibodies/tbk1-nak-d1b4-rabbit-mab/3504
Total RNAPII (F-12) https://www.scbt.com/p/pol-ii-antibody-f-12
gamma H2AX https://www.cellsignal.com/products/primary-antibodies/phospho-histone-h2a-x-ser139-antibody/2577
PCNA https://www.abcam.com/products/primary-antibodies/pcna-antibody-epr3821-ab92552.html
RAD9 https://www.thermofisher.com/antibody/product/RAD9-Antibody-Polyclonal/PA5-21275
RNAPII phospho Ser5 https://www.biolegend.com/fr-lu/products/purified-anti-rna-polymerase-ii-antibody-11544
CTR9 https://www.novusbio.com/products/ctr9-antibody_nb100-68205
Total RNAPII (A-10) https://www.scbt.com/p/pol-ii-antibody-a-10
SPT6 https://www.novusbio.com/products/spt6-antibody_nb100-2582
SPT5 https://www.scbt.com/p/spt5-antibody-d-3
RNAPII phosphor-Ser2 https://www.abcam.com/products/primary-antibodies/rna-polymerase-ii-ctd-repeat-ysptsps-phospho-s2-antibody-ab5095.html
SPT4 https://www.cellsignal.com/products/primary-antibodies/spt4-d3p2w-rabbit-mab/64828
NIPBL https://www.scbt.com/p/nipbl-antibody-c-9
MRE11 https://www.abcam.com/products/primary-antibodies/mre11-antibody-epr21027-chip-grade-ab208020.html
CHK1 (phosphoSer345) https://www.cellsignal.com/products/primary-antibodies/phospho-chk1-ser345-133d3-rabbit-mab/2348
RAD50 https://www.abcam.com/products/primary-antibodies/rad50-antibody-epr20968-chip-grade-ab208019.html
ATR https://www.thermofisher.com/antibody/product/ATR-Antibody-Polyclonal/A300-138A
CHK1 https://www.scbt.com/p/chk1-antibody-fl-476
FANCD2 https://www.abcam.com/products/primary-antibodies/fancd2-antibody-epr2302-ab108928.html
HUWE1 https://www.abcam.com/products/primary-antibodies/huwe1mule-antibody-ab70161.html
RNF20 https://www.cellsignal.com/products/primary-antibodies/rnf20-d6e10-xp-rabbit-mab/11974
Ubiquityl-Histone H2B (Lys120) https://www.cellsignal.com/products/primary-antibodies/ubiquityl-histone-h2b-lys120-d11-xp-rabbit-mab/5546
histone H2B https://www.abcam.com/products/primary-antibodies/histone-h2b-antibody-chip-grade-ab1790.html
CD45 https://www.biolegend.com/fr-fr/soluble-mhc/brilliant-violet-510-anti-mouse-cd45-antibody-7995?GroupID=BLG1932
CD3e https://www.biolegend.com/de-de/search-results/alexa-fluor-700-anti-mouse-cd3epsilon-antibody-13779?GroupID=BLG15684
PD-1/CD279 https://www.biolegend.com/de-de/search-results/brilliant-violet-421-anti-mouse-cd279-pd-1-antibody-7330?GroupID=BLG7927
CD11b https://www.biolegend.com/ja-jp/products/pe-anti-mouse-human-cd11b-antibody-349
CD11c https://www.biolegend.com/de-de/products/alexa-fluor-647-anti-mouse-cd11c-antibody-2703?GroupID=BLG11937
CD4 https://www.biolegend.com/fr-ch/products/brilliant-violet-650-anti-mouse-cd4-antibody-16780?GroupID=BLG4211
CD4 https://www.biolegend.com/fr-ch/products/brilliant-violet-605-anti-mouse-cd4-antibody-10708?GroupID=BLG4745
CD8 https://www.biolegend.com/fr-fr/products/fitc-anti-mouse-cd8a-antibody-153
CD8 https://www.biolegend.com/fr-lu/cell-health/pe-anti-mouse-cd8a-antibody-155?GroupID=BLG2559
CTLA-4 https://www.biolegend.com/fr-lu/products/pe-anti-mouse-cd152-antibody-516
CD45R/B220 https://www.biolegend.com/en-us/search-results/pe-dazzle-594-anti-mouse-human-cd45r-b220-antibody-10188?GroupID=GROUP658
Ly6G https://www.biolegend.com/en-us/products/percp-cyanine5-5-anti-mouse-ly-6g-antibody-6116?GroupID=BLG7234
Ly6C https://www.biolegend.com/nl-nl/products/alexa-fluor-700-anti-mouse-ly-6c-antibody-6757?GroupID=BLG7242
anti-F4/80 https://www.biolegend.com/nl-be/products/brilliant-violet-421-anti-mouse-f4-80-antibody-7199
Podoplanin https://www.biolegend.com/de-at/products/apc-anti-mouse-podoplanin-antibody-6656?GroupID=BLG5772
Ep-CAM https://www.biolegend.com/en-ie/products/brilliant-violet-421-anti-mouse-cd326-ep-cam-antibody-9964

E-Cadherin https://www.biolegend.com/de-at/products/pe-dazzle-594-anti-mouse-human-cd324-e-cadherin-antibody-16386
CTLA-4 https://www.biolegend.com/en-ie/products/percp-cyanine5-5-anti-mouse-cd152-antibody-10451?GroupID=BLG10448
CD206 https://www.biolegend.com/ja-jp/products/brilliant-violet-650-anti-mouse-cd206-mmr-antibody-8842
CD31 https://www.thermofisher.com/antibody/product/CD31-PECAM-1-Antibody-clone-390-Monoclonal/25-0311-82
CD44 https://www.thermofisher.com/antibody/product/CD44-Antibody-clone-IM7-Monoclonal/64-0441-82
MHCII https://www.thermofisher.com/antibody/product/MHC-Class-II-I-A-I-E-Antibody-clone-M5-114-15-2-Monoclonal/63-5321-82
PDGFRa https://www.thermofisher.com/antibody/product/CD140a-PDGFRA-Antibody-clone-APA5-Monoclonal/67-1401-82
H-2Db https://www.thermofisher.com/antibody/product/H-2Db-Antibody-clone-B22-249-R1-Monoclonal/MA5-17992
H-2Kb https://bioxcell.com/invivomab-anti-mouse-mhc-class-i-h-2kb
ECL anti-mouse IgG horseradish peroxidase https://www.sigmaaldrich.com/DE/en/product/sigma/gena9311ml
ECL anti-rabbit IgG horseradish peroxidase Sigma-Aldrich https://www.sigmaaldrich.com/DE/en/product/sigma/gena9341ml
Alexa Fluor® 647 Goat anti-rabbit IgG https://www.thermofisher.com/antibody/product/Goat-anti-Rabbit-IgG-H-L-Cross-Adsorbed-Secondary-Antibody-Polyclonal/A-21244
Alexa Fluor® 488 Goat anti-rabbit IgG https://www.thermofisher.com/antibody/product/Goat-anti-Rabbit-IgG-H-L-Cross-Adsorbed-Secondary-Antibody-Polyclonal/A-11008

Eukaryotic cell lines

Policy information about cell lines and Sex and Gender in Research

Cell line source(s)

KPC (established from a tumor of a male mouse) Jens Sieveke (Jung LA et al., 2017, Oncogene, DOI:10.1038/onc.2016.354, Krenz B et al., 2021, Cancer research, DOI:10.1158/0008-5472.CAN-21-1677).
U2OS ATCC CVCL_0042
HEK293TN ATCC CVCL_UL49
p53-mutant and p16ink4a(Cdkn2a)-deficient murine PDAC cell lines (established from tumors of male mice) were provided by Dieter Saur.
Panc-1 and PA-TU-8988T human PDAC cell lines were provided by Mathias Rosenfeldt.

Authentication

Authentication of the human cell lines used was done by STR profile testing, working according to the human cell line authentication standard published by ANSI. KPC cells were validated by RNA sequencing.

Mycoplasma contamination

All cell lines routinely tested negative for mycoplasma contamination.

Commonly misidentified lines
(See ICLAC register)

None of the used cell lines is listed in the ICLAC register.

Animals and other research organisms

Policy information about studies involving animals; ARRIVE guidelines recommended for reporting animal research, and Sex and Gender in Research

Laboratory animals

C57BL/6J (bred at the biocentre Wuerzburg) and NOD-Rag2-IL2rgTm1/Rj mice (NRG, ordered by Janvier Labs) were used. KPC cells were injected in 5-12 weeks old male mice. The mice were treated prophylactically with painkillers for three days.
C57BL/6J mice were kept in type II-L cages, NRG mice were kept in individually ventilated cages (IVC).
One week after injection tumor cells (luciferase positive) were measured by bioimaging (IVIS). On the basis of the measurement, the experimental groups were divided in such a way that groups with approximately equally strong signals were created. The treatment started after the division of the groups.
There is a 12-hour light/dark cycle in the animal housing (7 am to 7 pm light). The temperature in the animal housing rooms is between 20°C and 24°C and the relative humidity is between 45% and 65%.

Wild animals

No wild animals were used

Reporting on sex

To avoid immunological rejection, male mice were used in our experiments because the KPC cells we used for orthotopic transplantation also came from a male mouse.

Field-collected samples

No field-collected samples were used

Ethics oversight

The application was approved by the Government of Lower Franconia (Regierung von Unterfranken) under the number RUF-55.2.2-2532-2-1419 and RUF-55.2.2-2532-148.

Note that full information on the approval of the study protocol must also be provided in the manuscript.

Plants

Seed stocks

Report on the source of all seed stocks or other plant material used. If applicable, state the seed stock centre and catalogue number. If plant specimens were collected from the field, describe the collection location, date and sampling procedures.

Novel plant genotypes

Describe the methods by which all novel plant genotypes were produced. This includes those generated by transgenic approaches, gene editing, chemical/radiation-based mutagenesis and hybridization. For transgenic lines, describe the transformation method, the number of independent lines analyzed and the generation upon which experiments were performed. For gene-edited lines, describe the editor used, the endogenous sequence targeted for editing, the targeting guide RNA sequence (if applicable) and how the editor was applied.

Authentication

Describe any authentication procedures for each seed stock used or novel genotype generated. Describe any experiments used to assess the effect of a mutation and, where applicable, how potential secondary effects (e.g. second site T-DNA insertions, mosiacism, off-target gene editing) were examined.

Methodology

Replicates

RNA, 4-sU-seq. and BLISS experiments were done in biological triplicats (n=3)
CUTnRun and ChIP-seq experiment were performed twice (n=2)
We were not able to generate a sequencing library for ChIP-seq. of CTR9 upon CTR9 depletion for replicate number 2.

Sequencing depth

GSM7136972 ChIP-seq RNAPII shCTR9 EtOH 20805257
GSM7136973 ChIP-seq RNAPII shCTR9 Dox 24914291
GSM7136974 ChIP-seq CTR9 shCTR9 EtOH 32562598
GSM7136975 ChIP-seq CTR9 shCTR9 Dox 29934091
GSM7136977 ChIP-seq SPT5 shCTR9 EtOH 68139390
GSM7136978 ChIP-seq SPT5 shCTR9 Dox 30581236
GSM7136979 ChIP-seq SPT6 shCTR9 EtOH 43451061
GSM7136980 ChIP-seq SPT6 shCTR9 Dox 48770164
GSM7136981 ChIP-seq Input merged 32061360
GSM7136982 CUT&RUN MYC shCTR9 EtOH 12979494
GSM7136983 CUT&RUN MYC shCTR9 Dox 12760475
GSM7136985 CUT&RUN MYC shMYC EtOH 13866457
GSM7136986 CUT&RUN MYC shMYC Dox 11711172
GSM7136987 CUT&RUN IgG shCTR9 EtOH 12728886
GSM7137684 RNA-seq shLuciferase Dox rep1 15889288
GSM7137686 RNA-seq shLuciferase Dox rep2 17011297
GSM7137687 RNA-seq shLuciferase Dox rep3 17196078
GSM7137688 RNA-seq shCTR9 Dox rep1 15672953
GSM7137689 RNA-seq shCTR9 Dox rep2 15812453
GSM7137690 RNA-seq shCTR9 Dox rep3 29815450
GSM7137691 RNA-seq shMYC Dox rep1 15641959
GSM7137693 RNA-seq shMYC Dox rep2 15781891
GSM7137694 RNA-seq shMYC Dox rep3 14605517
GSM7137695 RNA-seq shCDC73 Dox rep1 15559308
GSM7137696 RNA-seq shCDC73 Dox rep2 15278453
GSM7137697 RNA-seq shCDC73 Dox rep3 16917746
GSM7138383 BLISS shMYC EtOH rep1 13153492
GSM7138384 BLISS shMYC EtOH rep2 12606928
GSM7138385 BLISS shMYC EtOH rep3 14530140
GSM7138386 BLISS shMYC Dox rep1 3012410
GSM7138387 BLISS shMYC Dox rep2 3505034
GSM7138389 BLISS shMYC Dox rep3 3183610
GSM7138390 BLISS shMYC EtOH AZD6738 rep1 10399042
GSM7138391 BLISS shMYC EtOH AZD6738 rep2 10741877
GSM7138392 BLISS shMYC EtOH AZD6738 rep3 13548451
GSM7138393 BLISS shMYC Dox AZD6738 rep1 6174196
GSM7138394 BLISS shMYC Dox AZD6738 rep2 6898909
GSM7138395 BLISS shMYC Dox AZD6738 rep3 6846641
GSM7794505 4sU-seq shLuciferase Dox rep1 49110602
GSM7794506 4sU-seq shLuciferase Dox rep2 59237642
GSM7794508 4sU-seq shLuciferase Dox rep3 52624837
GSM7794509 4sU shCTR9 Dox rep1 49434030
GSM7794510 4sU shCTR9 Dox rep2 48223861
GSM7794511 4sU shCTR9 Dox rep3 44538305
GSM7794514 ChIP-seq RNAPII shCTR9 EtOH rep2 38863168
GSM7794515 ChIP-seq RNAPII shCTR9 Dox rep2 41105880
GSM7794516 ChIP-seq SPT5 shCTR9 EtOH rep2 40619586
GSM7794517 ChIP-seq SPT5 shCTR9 Dox rep2 38852347
GSM7794518 ChIP-seq SPT6 shCTR9 EtOH rep2 34384144
GSM7794519 ChIP-seq SPT6 shCTR9 Dox rep2 32347439
GSM7794520 ChIP-seq Input merged rep2 33865542
GSM7794521 ChIP-seq CTR9 shCTR9 EtOH rep2 22995082
GSM7794522 CUT&RUN MYC shCTR9 EtOH rep2 12028709
GSM7794523 CUT&RUN MYC shCTR9 Dox rep2 13056057

Antibodies

RNAPII, Santa Cruz Biotechnology, sc-17798, A10, Lot: J1921
SPT5, Santa Cruz Biotechnology, sc-133217, Lot: E2821
SPT6, Novus, NB100-2582, Lot: A2
CTR9, Bethyl, A301-395A, Lot:301-395A-4

Peak calling parameters

ChIP-sequenced libraries were mapped separately to mouse mm10 genome and human hg19 (spike-in) using Bowtie 2 v2.3.4.1 with default parameters. ChIP-Rx spike-in normalization was performed by calculating spike-in normalization factor, by dividing the number of mapped reads from the spike-in of the smallest sample by the number of mapped reads from the spike-in of each sample. For each sample, this factor was multiplied by the number of reads that mapped to the mouse genome. Samtools was used to manipulate the bam files (indexing and subsampling).

ChIP-seq

Data deposition

☒ Confirm that both raw and final processed data have been deposited in a public database such as GEO.

☐ Confirm that you have deposited or provided access to graph files (e.g. BED files) for the called peaks.

Data access links<br>*May remain private before publication.*	GSE228800
Files in database submission	GSM7136972 ChIP-seq RNAPII shCTR9 EtOH<br>GSM7136973 ChIP-seq RNAPII shCTR9 Dox<br>GSM7136974 ChIP-seq CTR9 shCTR9 EtOH<br>GSM7136975 ChIP-seq CTR9 shCTR9 Dox<br>GSM7136977 ChIP-seq SPT5 shCTR9 EtOH<br>GSM7136978 ChIP-seq SPT5 shCTR9 Dox<br>GSM7136979 ChIP-seq SPT6 shCTR9 EtOH<br>GSM7136980 ChIP-seq SPT6 shCTR9 Dox<br>GSM7136981 ChIP-seq Input merged<br>GSM7136982 CUT&RUN MYC shCTR9 EtOH<br>GSM7136983 CUT&RUN MYC shCTR9 Dox<br>GSM7136985 CUT&RUN MYC shMYC EtOH<br>GSM7136986 CUT&RUN MYC shMYC Dox<br>GSM7136987 CUT&RUN IgG shCTR9 EtOH<br>GSM7137684 RNA-seq shLuciferase Dox rep1<br>GSM7137686 RNA-seq shLuciferase Dox rep2<br>GSM7137687 RNA-seq shLuciferase Dox rep3<br>GSM7137688 RNA-seq shCTR9 Dox rep1<br>GSM7137689 RNA-seq shCTR9 Dox rep2<br>GSM7137690 RNA-seq shCTR9 Dox rep3<br>GSM7137691 RNA-seq shMYC Dox rep1<br>GSM7137693 RNA-seq shMYC Dox rep2<br>GSM7137694 RNA-seq shMYC Dox rep3<br>GSM7137695 RNA-seq shCDC73 Dox rep1<br>GSM7137696 RNA-seq shCDC73 Dox rep2<br>GSM7137697 RNA-seq shCDC73 Dox rep3<br>GSM7138383 BLISS shMYC EtOH rep1<br>GSM7138384 BLISS shMYC EtOH rep2<br>GSM7138385 BLISS shMYC EtOH rep3<br>GSM7138386 BLISS shMYC Dox rep1<br>GSM7138387 BLISS shMYC Dox rep2<br>GSM7138389 BLISS shMYC Dox rep3<br>GSM7138390 BLISS shMYC EtOH AZD6738 rep1<br>GSM7138391 BLISS shMYC EtOH AZD6738 rep2<br>GSM7138392 BLISS shMYC EtOH AZD6738 rep3<br>GSM7138393 BLISS shMYC Dox AZD6738 rep1<br>GSM7138394 BLISS shMYC Dox AZD6738 rep2<br>GSM7138395 BLISS shMYC Dox AZD6738 rep3<br>GSM7794505 4sU-seq shLuciferase Dox rep1<br>GSM7794506 4sU-seq shLuciferase Dox rep2<br>GSM7794508 4sU-seq shLuciferase Dox rep3<br>GSM7794509 4sU shCTR9 Dox rep1<br>GSM7794510 4sU shCTR9 Dox rep2<br>GSM7794511 4sU shCTR9 Dox rep3<br>GSM7794514 ChIP-seq RNAPII shCTR9 EtOH rep2<br>GSM7794515 ChIP-seq RNAPII shCTR9 Dox rep2<br>GSM7794516 ChIP-seq SPT5 shCTR9 EtOH rep2<br>GSM7794517 ChIP-seq SPT5 shCTR9 Dox rep2<br>GSM7794518 ChIP-seq SPT6 shCTR9 EtOH rep2<br>GSM7794519 ChIP-seq SPT6 shCTR9 Dox rep2<br>GSM7794520 ChIP-seq Input merged rep2<br>GSM7794521 ChIP-seq CTR9 shCTR9 EtOH rep2<br>GSM7794522 CUT&RUN MYC shCTR9 EtOH rep2<br>GSM7794523 CUT&RUN MYC shCTR9 Dox rep2
Genome browser session<br>(e.g. UCSC)	Graphical files can be downloaded from GEO
Data quality	Sequencing quality of FASTQ files was checked using FastQC.
Software	Reads were mapped to the mouse reference genome (mm10) with Bowtie2 v2.3.4.1. The BAM files were normalized (subsampled) to read counts and indexed using SAMtools v1.9. To visualize read alignments on the Integrated Genome Browser v9.1.10, BAM files were converted to bigwig files using Deeptools with no further normalization and a bin size of 10 bases. Heat maps were generated by applying the computeMatrix operation to all expressed genes followed by plotHeatMap function in DeepTools v3.5.1. Metagene

plot of MYC CUT&RUN was generated by applying the computeMatrix operation to all expressed genes (Centered around TSS) followed by plotProfile function in DeepTools v3.5.1. For the mRNA-seq, Reads per gene were counted using the featureCounts function from the R package Rsubread v2.12.2. Go terms were identified using GOstats v2.64.0 and enrichR v3.1 packages in R version v4.2.2.

Flow Cytometry

Plots

Confirm that:

☒ The axis labels state the marker and fluorochrome used (e.g. CD4-FITC).

☒ The axis scales are clearly visible. Include numbers along axes only for bottom left plot of group (a 'group' is an analysis of identical markers).

☒ All plots are contour plots with outliers or pseudocolor plots.

☒ A numerical value for number of cells or percentage (with statistics) is provided.

Methodology

Sample preparation	Extracted tumors were mechanically disrupted and digested in a 500-µL mixture of 1 mg/mL collagenase A and D, trypsin inhibitor from glycine max and 0.4 mg/mL DNase I in PBS first at 22°C for 45 minutes and then at 37°C for 15 minutes with 1000 rpm rotation in a thermo-mixer . EDTA was then added to a final concentration of 10 mmol/L, whereafter the cells were passed through a 70-µm mesh prior to immunostaining. Cells were stained with fixable viability dye eFluor780 (1:1000, eBioscience, #65-2860-40) for 15 min at 4°C, blocked with anti-mouse TruStain FcX™ for 5 min at 4°C and stained for 30 min at 4°C with combinations fluorescently conjugated antibodies. Anti-CD206 BV650 was added after surface staining was completed and after fixation-permeabilization using the FoxP3/Transcription Factor Fixation/Permeabilization Kit and the manufacturer's guidelines were followed.
Instrument	All flow cytometry was performed on an Attune NxT (Thermo Fisher) analyzer.
Software	Offline analysis was performed with FlowJo software (Treestar, version 10.9).
Cell population abundance	About 90% of all measured living cells are CD45-positive what is comparable with flow cytometry experiments we have previously performed in the same transplant model. About 40-50% of CD45+ cells where T cells. Most CD3+ T cells are either CD4 or CD8 positive (80-90%). Dendritic cells only make a small proportion of the immune infiltrate.
Gating strategy	Gating of lymphocytes, singlets, living cells was performed as shown in Supplementary Figure 8b. Supplementary 8b also includes gating for CD4, CD4, CD8, CD45, CD11b and CD11c.

☒ Tick this box to confirm that a figure exemplifying the gating strategy is provided in the Supplementary Information.
